# Crystallographic evidence for global aromaticity in the di-anion and tetra-anion of a cyclophane hydrocarbon†

**DOI:** 10.1039/d3sc04251k

**Published:** 2023-09-20

**Authors:** Wojciech Stawski, Yikun Zhu, Zheng Wei, Marina A. Petrukhina, Harry L. Anderson

**Affiliations:** a Department of Chemistry, University at Albany, State University of New York Albany NY 12222 USA mpetrukhina@albany.edu; b Department of Chemistry, University of Oxford, Chemistry Research Laboratory Oxford OX1 3TA UK harry.anderson@chem.ox.ac.uk

## Abstract

[2_4_]Paracyclophanetetraene is a classic example of a macrocyclic hydrocarbon that becomes globally aromatic on reduction to the di-anion, and switches to globally anti-aromatic in the tetra-anion. This redox activity makes it promising as an electrode material for batteries. Here, we report the solid-state structures of the di- and tetra-anions of this cyclophane, in several coordination environments. The changes in bond length on reduction yield insights into the global aromaticity of the di-anion (26π electrons), and anti-aromaticity of the tetra-anion (28π electrons), that were previously deduced from NMR spectra of species generated *in situ*. The experimental geometries of the aromatic di-anion and anti-aromatic tetra-anion from X-ray crystallographic data match well with gas-phase calculated structures, and reproduce the low symmetry expected in the anti-aromatic ring. Comparison of coordinated and naked anions confirms that metal coordination has little effect on the bond lengths. The UV-vis-NIR absorption spectra show a sharp intense peak at 878 nm for the di-anion, whereas the tetra-anion gives a broad spectrum typical of an anti-aromatic system.

## Abstract 2

Anionic forms of a macrocyclic cyclophane were crystallized by treating the neutral hydrocarbon with alkali metals (Li, Na and K). The di-anions show decreased bond-length alternation, consistent with global aromaticity, whereas the anti-aromatic tetra-anion has a low-symmetry geometry.

## Graphical abstract

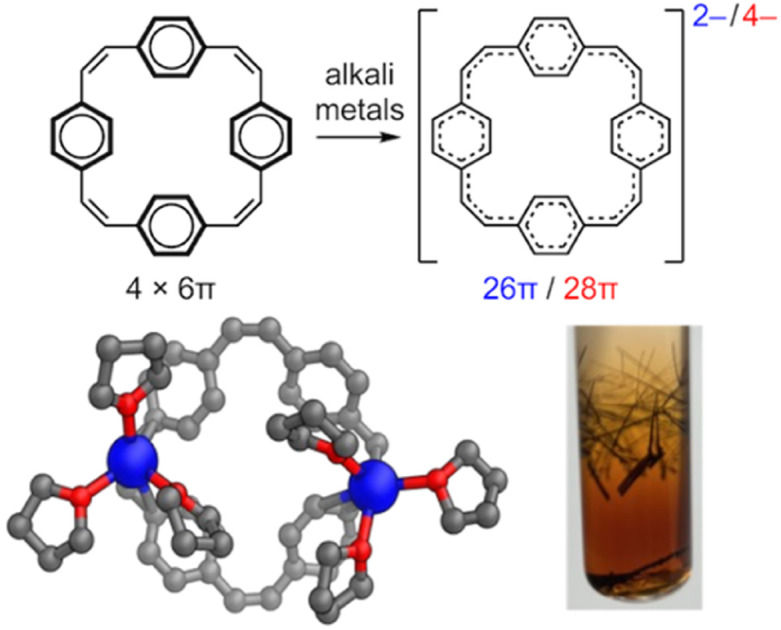

## Introduction

**Fig. 1:**
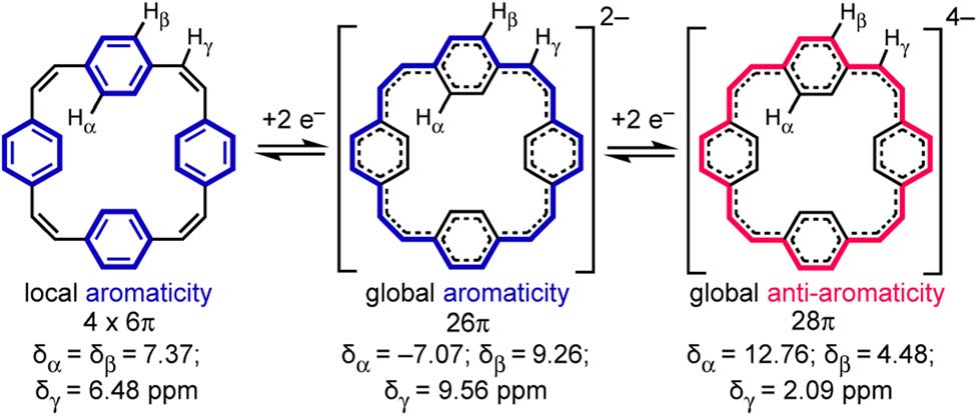
Reduction of neutral 1 (PCT) to the di- and tetra-anion, showing the number of π electrons in the delocalization pathways and ^1^H NMR chemical shifts in THF-*d*_8_, −80 °C (data from ref. 3a).

Reduction of π-conjugated hydrocarbons with alkali metals can profoundly alter their electronic structures and properties.^1^ [2_4_]Paracyclophanetetraene 1 (PCT, Fig. 1) is one of the first macrocyclic hydrocarbons reported to exhibit global electronic delocalization upon reduction.^2,3^ The electronic structure of the nonplanar neutral form is described by four localized benzene Clar sextets, separated by *cis*-vinylene bridges with purely olefinic character. Adding two or four electrons generates a delocalization pathway leading to global aromaticity (di-anion: 26π e^−^) or anti-aromaticity (tetra-anion: 28π e^−^), manifested by large chemical shift differences between the inner and outer core protons in the ^1^H NMR spectra (H_α_ and H_β_, Fig. 1).^3^ It has been questioned whether global aromaticity can be reliably assigned in large macrocycles based purely on NMR data,^4^ which motivated us to check for structural evidence from global aromaticity. The redox-based aromaticity switching in 1 may make it a suitable material for storing energy in batteries,^5^ and several derivatives of 1 have been synthesized for this application.^6^ It is desirable to know the solid-state structure of anions involved in these processes, and to understand their coordination chemistry with alkali metal cations. Although the redox behavior of 1 has been known for over 40 years, the crystal structures of the reduced species have not been reported. We decided to take up this challenge and characterize them in the solid state.

## Results and discussion

**Fig. 2:**
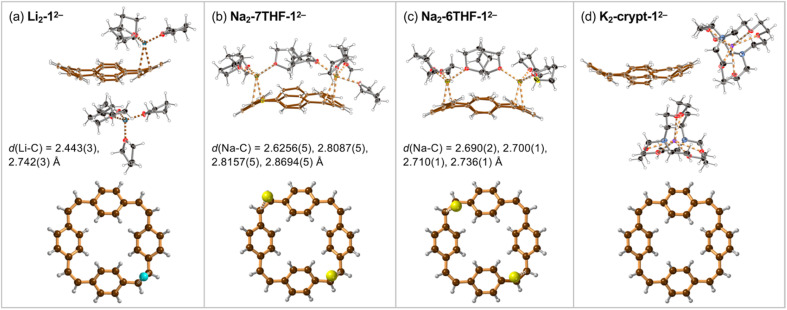
Single crystal X-ray structures of (a) Li_2_-1^2−^, (b) Na_2_-7THF-1^2−^, (c) Na_2_-6THF-1^2−^ and (d) K_2_-crypt-1^2−^ (thermal ellipsoids: 40% probability). In each case, the lower image is a projection perpendicular to the mean plane of the PCT di-anion, with coordinated metal cations. The shortest metal-carbon distances are indicated by dashed lines and listed. Solvent molecules and minor disorder components are omitted for clarity. Color code: Li: sky-blue; Na: yellow; K: purple; N: blue; O: red; C: black or brown; H: pale gray.

Crystals of the PCT salts were grown by reducing the cyclophane with alkali metals in THF, followed by layered addition of hexane. Plate-like crystals of Li and Na salts of the di-anion 1^2−^ appeared after few hours. The lithium salt of 1^2−^ crystallized with one lithium cation coordinated to a vinylene bridge and the second Li^+^ solvent-separated (Fig. 2a). The molecular formula of this product can be written as [Li^+^(THF)_4_][{Li^+^(THF)_3_}(1^2−^)] and we use the shortened Li_2_-1^2−^ (and similarly for other products) throughout the text.

Reaction of 1 with sodium provided two types of crystals of close–contact complexes, Na_2_-6THF-1^2−^ and Na_2_-7THF-1^2−^ (Fig. 2b and c). In Na_2_-6THF-1^2−^, described more accurately as [Na^+^(THF)_3_]_2_[1^2−^], both counterions coordinate to three THF molecules and bind to two C=C bridges. Despite the different numbers of coordinated THF molecules in Na_2_-7THF-1^2−^, with the more descriptive formula of [Na^+^(THF)_3_][Na^+^(THF)_4_][1^2−^], both Na^+^ cations also bind C=C bridges, as in Na_2_-6THF-1^2−^. In both cases, the cations are located on the same side of the hydrocarbon di-anion, on the opposite C=C bridges.

In order to check the impact of metal coordination on electronic delocalization, 18-crown-6 and [2.2.2]cryptand were added to the reaction mixtures with sodium and potassium, respectively. Two solvent-separated products were isolated. [K^+^(cryptand)]_2_[1^2−^], abbreviated as K_2_-crypt-1^2−^, features two cryptand-bound cations, separating them from the hydrocarbon di-anion (Fig. 2d). Similarly, Na_2_-crown-1^2−^, with the detailed formula of [Na^+^(18-crown-6)(THF)_2_]_2_[1^2−^], contains 1^2−^ di-anions solvent-separated from the cations, which coordinate to the crown ether and THF (ESI, Fig. S10†).

**Fig. 3:**
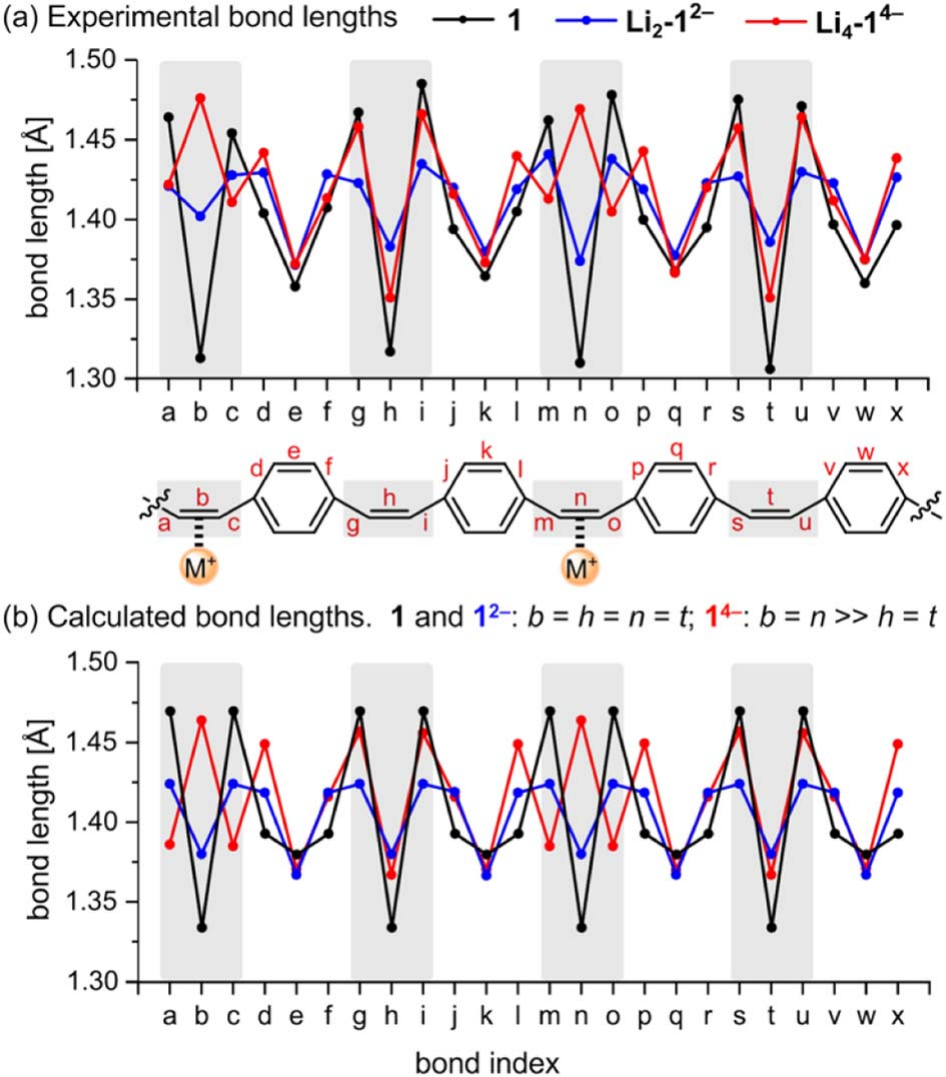
Comparison of (a) crystallographic C–C bond lengths in 1, Li_2_-1^2−^ and Li_4_-1^4−^, and (b) calculated bond lengths for gas-phase 1, 1^2−^ and 1^4−^ from DFT (CAM-B3LYP/def2-TZVP). Bonds in the vinylene bridges are marked in gray. Lengths for the *para*-phenylene units (*e.g. d*–*f*) are averages for inner and outer bonds.

**Fig. 4:**
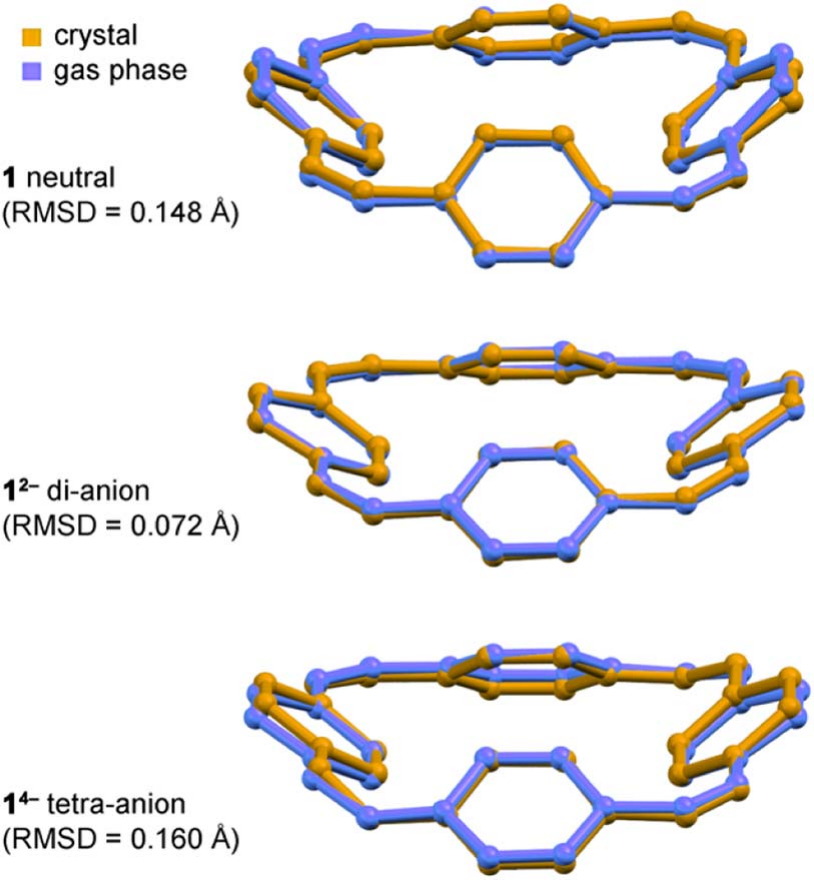
Overlay of crystallographic (orange) and calculated gas-phase (blue) structures of neutral 1, Li_2_-1^2−^ and Li_4_-1^4−^, with RMSDs between experimental and calculated structures. Note that the Li^+^ cations were not included in the gas phase DFT calculations. Calculations: CAM-B3LYP def2-TZVP.

The geometries of the 1^2−^ di-anions in all five crystal structures are remarkably similar, and they closely resemble the geometry of gas-phase 1^2−^ predicted by density functional theory (DFT), as shown by the patterns of bond lengths plotted in Fig. 3 (see also Fig. S17† for comparison of the crystallographic bond lengths in 1^2−^ di-anions) and by the overlayed projections in Fig. 4 (solid-state structure in orange; gas-phase structure in blue). Reduction of neutral 1 to 1^2−^ results in a global decrease in bond-length alternation around the macrocycle (Fig. 3, black and blue traces). The changes are most striking in the vinylene bridges. For example, in Li_2_-1^2−^, the single C=C bond coordinated to the Li^+^ cation is most elongated (bond *b*; 1.402(2) Å) compared with 1.312(5) Å in the neutral 1, but the other three remote C=C bonds are almost as elongated (*h*: 1.386(2) Å; *n*: 1.374(2) Å, *t*: 1.383(2) Å) reflecting the global change in electronic structure as the whole system becomes aromatic with a circuit of 26π-electrons. The elongation of the C=C double bonds (*b*, *h*, *n*, *t*) is accompanied by a contraction of the C–C single bonds (*a*, *c*, *g*, *i*, *m*, *o*, *s*, *u*), corresponding to a reduction in bond-length alternation.

We performed DFT calculations on isolated anions in the gas phase in order to compare the experimental bond lengths with the theoretical values (CAM-B3LYP/def2-TZVP; Fig. 3).^5,7^ In the optimized structure of di-anion, the length of the middle bond in the C=C bridge (*b*) is 1.380 Å whereas the bonds linking it to the *para*-phenylene rings (*a*) are 1.424 Å (Fig. 3b). These values are close to the experimentally determined lengths for bridges not involved in metal binding, with the general trend that coordination to metal cations increases the length of the C=C bond by *ca.* 0.01–0.02 Å, depending on the metal. Comparison of the direct-contacted and solvent-separated anions shows that metal coordination only slightly perturbs the effect of electronic delocalization over the whole molecule.

The global electronic delocalization in the di-anion can be evaluated using the harmonic oscillator model of aromaticity (HOMA), which compares bond lengths with those in benzene (see ESI, Section 7†).^8^ The vinylene bridges of neutral 1 give a HOMA value of −0.66 (non-aromatic), whereas the five crystal structures of 1^2−^ salts give HOMA values in the range 0.66–0.73 (compared with an ideal value of 1.00 for fully aromatic).

In all the structures of the 1^2−^ di-anion, the torsion angles in the linkers (defined by the coordinates of ethylene bridges and the two carbon atoms directly connected to them) increase substantially upon reduction, consistently with the theoretical predictions and with a reduction in bond order (see ESI, Section 3.2†). The whole C_32_H_24_ framework generally becomes flatter on reduction to the di-anion, reflecting greater π-delocalization around the macrocycle. Thus the root-mean-square deviation (RMSD) of the 32 carbon atoms from a plane decreases from 0.527 Å in neutral 1 to 0.395 Å in 1^2−^ in the DFT geometries; the experimental RMSDs are 0.411 Å in Li_2_-1^2−^, 0.501 Å in Na_2_-7THF-1^2−^, 0.452 Å in Na_2_-6THF-1^2−^ and 0.462 Å in K_2_-crypt-1^2−^, compared with 0.471 Å in neutral 1.^2*a*^

**Fig. 5:**
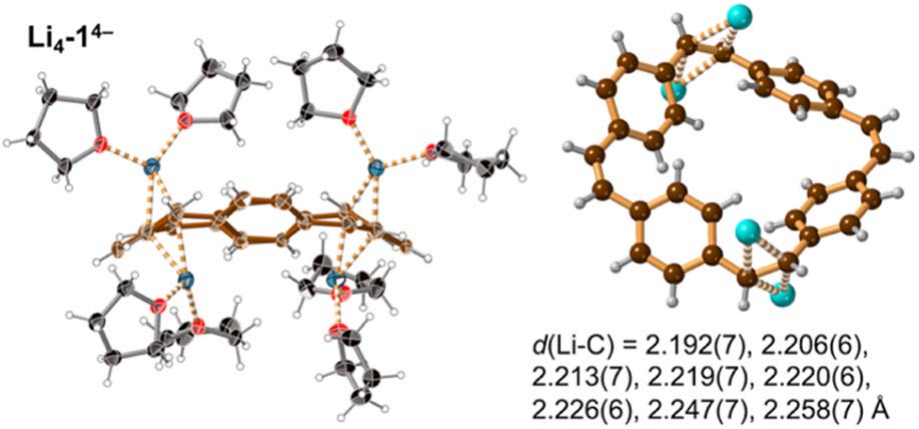
Single crystal X-ray structure of Li_4_-1^4−^. Thermal ellipsoids at 40% probability level and ball-and-stick view. Minor disorder components are omitted for clarity. Color code as for Fig. 2. The shortest eight Li⋯C distances are indicated by dashed lines and listed.

Prolonged contact of 1 with lithium metal in THF provided a tetra-reduced product [Li^+^(THF)_2_]_4_[1^4−^] (Li_4_-1^4−^, Fig. 5). The lithium cations coordinate to the opposite C2 linkers, two to each vinylene bridge. Each cation is additionally coordinated to two THF molecules. The 1^4−^ macrocycle exhibits stronger bond-length alternation than 1^2−^ (Fig. 3a, red trace). The C2 bridges that are coordinated to Li^+^ cations (*b* and *n*) are remarkably elongated (1.469(4) and 1.476(4) Å) compared with the C=C bond length in neutral 1 (1.312(4) Å). The C2 bridges that are not coordinated by lithium cations (*h* and *t*) are slightly elongated (1.350(4) and 1.351(4) Å). Bridges involved in coordination to metal centers are twisted, with torsion angles (48.3(3)/51.5(3)° *vs.* 5.3(3)/4.4(3)° in 1; see ESI†).

The pattern of experimental bond lengths and torsion angles in the 1^4−^ tetra-anion is similar to that in the gas-phase from DFT calculations (Fig. 3b), revealing that the substantial elongation of the two C=C bridges involved in binding to lithium (*b* and *n*) is not a result of metal coordination. The gas-phase geometry has two long vinylene bridges (*b* and *n*), which have higher negative charge (Fig. S42†). The less symmetrical distribution of bond lengths in 1^4−^ is evidently a consequence of its anti-aromatic electronic structure.

Lithium is the only metal which provided a solution of a pure tetra-anion, which parallels results on polycyclic aromatic hydrocarbons.^9^ Analysis by ^1^H NMR spectroscopy shows that reduction with sodium yields a mixture of tetra-anion and di-anion (Fig. S25†), and only the di-anion crystallized from this mixture. Prolonged contact with the potassium resulted in a black precipitate, suggesting that the tetra-anion K^+^ salt is insoluble in THF. Indeed, the ^1^H NMR spectrum of the potassium di-anion salt was reported by Müllen *et al.*, without commenting on the tetra-anion.^3*b*^ Contact of a solution of 1 with rubidium or cesium metal causes immediate precipitation, even in the presence of secondary ligands. Addition of crown ethers or [2.2.2]cryptand stops reduction by sodium or potassium at the di-anion stage (for lithium as representative example, see Fig. S37†). When a solution of 1 was exposed to K in the presence of 18-crown-6 or [2.2.2]cryptand, the solution remained intensely orange, without any precipitate, indicating that it also stopped at the di-anion. We can therefore control the maximum available reduction state by adding a secondary ligand.

We recorded ^1^H NMR spectra of all the crystal samples dissolved in *d*_8_-THF. Previously, the only spectra reported were for lithium salts of 1^2−^ and 1^4−^ (Fig. 1),^3*a*^ and the potassium salt of 1^2−^, generated *in situ*.^3*b*^ All the ^1^H NMR spectra of the di-anionic species consist of three singlets, two at high chemical shift (outer protons, H_β_ and H_γ_, 9.2 to 9.6 ppm) and one at negative chemical shift (inner protons, H_α_ −6.8 to −7.1 ppm). The simplicity of the spectra implies that there is a rapid exchange between metal binding sites. Li_2_-1^2−^ and Na_2_-1^2−^ give sharp signals at −30 °C, whereas it is necessary to decrease the temperature to −80 °C to see partial splitting of two high frequency signals in Na_2_-crown-1^2−^ and K_2_-crypt-1^2−^, and at this temperature the solubility drops resulting in a poor signal-to-noise ratio. The chemical shifts are summarized in Table 1. As noted previously, the shielded inner proton is sensitive to the counter cation (H_α_: *δ*_H_ = −7.10 ppm at −30 °C for Li_2_-1^2−^, *vs. δ*_H_ = −6.94 ppm for Na_2_-1^2−^).^3*b*^ The ^7^Li NMR spectrum of Li_2_-1^2−^ recorded at −30 °C exhibits a peak at *δ*_Li_ = −3.02 ppm, indicating that binding to Li^+^ results in shielding of the cation by the π-system (*c.f. δ*_Li_ = 0 ppm for LiCl in *d*_8_-THF).

**Table 1:** ^1^H NMR chemical shifts for 1 and its anionic salts at a range of temperatures^a^

Compound	*T* (°C)	*δ*_α_ (ppm)	*δ*_β_ (ppm)	*δ*_γ_ (ppm)
1	25	7.32	7.32	6.42
	−30	7.44	7.44	6.39
Li_2_-1^2−^	25	−6.85	9.21	9.49
	−30	−7.10	9.25	9.54
Na_2_-1^2−^	−30	−6.94	9.27	9.57
Na_2_-crown-1^2−^	−90	−7.05	9.19	9.49
K_2_-crypt-1^2−^	−80	−6.76	9.23	9.46
Li_4_-1^4−^	25	12.39	4.59	2.30
	−30	13.76	4.19	1.80

a Spectra recorded in THF-*d*_8_ at 500 MHz; see Fig. 1 for definition of α, β and γ proton environments.

In the ^1^H NMR spectra of Li_4_-1^4−^ recorded at 25 °C, the trend is opposite to that in the di-anion, and the inner protons (H_α_) resonate at high chemical shift (12.39 ppm) whereas the outer protons (H_β_ and H_γ_) appear at low frequencies (2.30 and 4.59 ppm), due to the presence of a paratropic ring current.^3,10^ The ^7^Li NMR spectrum shows a broad peak at *δ*_Li_ = 0.25 ppm, implying that the tetra-anion is solvent-separated.

**Fig. 6:**
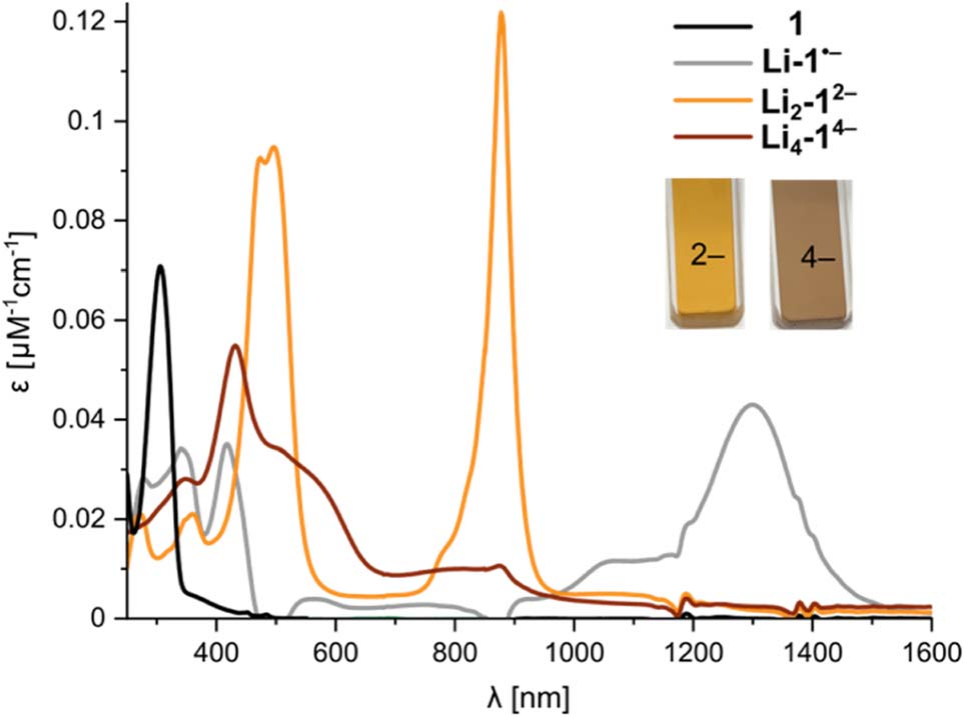
UV-vis-NIR spectra from *in situ* reduction of 1 with Li metal in THF at 25 °C, corresponding to the neutral macrocycle, radical anion, di-anion and tetra-anion (for details, see Fig. S18†).

Previous studies of PCT have not mentioned the spectacular color changes that accompany reduction, nor reported the UV-vis-NIR absorption spectra of the reduced species. A solution of neutral PCT in THF exhibits a band at 306 nm, with a shoulder reaching the visible region, resulting in a yellow color (Fig. 6). Reduction with lithium metal gives a bright orange solution, with the most red-shifted band at 878 nm, corresponding to the di-anion. Reduction occurs *via* a radical anion, with a broad band at 1300 nm, which was previously studied by EPR spectroscopy.^11^ Further reduction to the tetra-anion is characterized by isosbestic points, indicating that the intermediate trianion does not build up to significant concentrations. The tetra-anion has a rather featureless UV-vis-NIR absorption spectrum, as is characteristic of anti-aromatic species.^12^ The experimental absorption spectra agree moderately well with simulated spectra from TD-DFT calculations (CAM-B3LYP def2-TZVP) for the free anions in the gas phase (Fig. S43 and S44†). The UV-vis-NIR spectra of anions generated *in situ* match those recorded upon dissolving crystals (Fig. S22†). The type of counterion (Li^+^–K^+^) and the presence of crown ether or cryptand have negligible effect on the optical spectra, in line with the relatively small differences observed in ^1^H NMR spectra, confirming that the anions are solvent-separated in THF solution.

## Conclusions

Four features of the crystal structures of the 1^2−^ and 1^4−^ anions are remarkable:

### (a) Reduced bond-length alternation in the di-anion

**Fig. 7:**
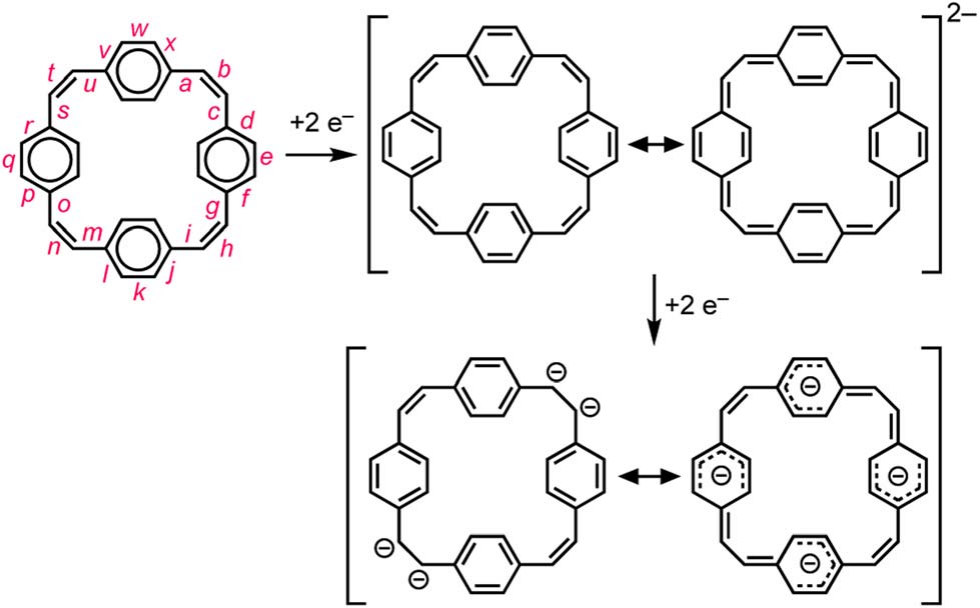
Schematic representation of the changes in delocalization revealed by the crystallographic molecular geometries. Reduction of the neutral cyclophane, with four localized Clar sextets, gives a globally delocalized aromatic di-anion. Further reduction gives a tetra-anion that can be viewed as the combination of four benzyl anions.

Global aromatic delocalization in the di-anion is revealed by the fact that the nominally single bonds (*a*, *c*, *g*, *i*, *m*, *o*, *s* and *u*) get shorter, while the nominally double bonds (*b*, *h*, *n* and *t*) get longer, on reduction of 1 to 1^2−^ (Fig. 3), as expected from a simple resonance scheme (Fig. 7). Local aromaticity in the benzene rings could not account for these changes in bond length.

### (b) Charge localization in the tetra-anion

Symmetry-breaking is a classical signature of anti-aromatic system (as illustrated by the *D*_2h_ symmetry of cyclobutadiene) and this effect is clearly evident in the crystallographic and calculated geometries of 1^4−^. It is most noticeable from the elongation of bonds *b* and *n*, the shortening of bonds *t* and *h*, and the elongation of bonds *g*, *i*, *s* and *u* (Fig. 3 and 6). Charge localization is confirmed experimentally by the locations of the counter ions in the crystal structures (near bonds *b* and *n*), and by the by gas-phase DFT Mulliken charge distribution (Fig. S42†). There must be some global anti-aromatic delocalization in 1^4−^ because the ^1^H NMR spectrum indicates a paratropic ring current, but it appears to be less delocalized than the di-anion.

### (c) Planarization

Text books list the formation of a planar π-system as one of the requirements for aromaticity. Thus it is noteworthy that the crystallographic RMSD of the 32 carbon atoms from a plane decreases from 0.471 Å in neutral 1 to 0.411 Å in Li_2_-1^2−^. This planarization is achieved through a reduction in the torsion angle between the vinylene and phenylene units, which enhances global delocalization. The corresponding RMSD of 1^4−^ is 0.492 Å reflecting its more distorted structure. The ^1^H NMR spectra of 1^2−^ and 1^4−^ provide further evidence of planarization. The fact that protons H_α_ and H_β_ give distinct resonances in the ^1^H NMR spectra of 1^2−^ and 1^4−^, but not for neutral 1, shows that reduction increases the barrier for phenylene rotation, which is related to the preference for a planar structure.

### (d) Match between solid-state and gas-phase geometries

All three features (a)–(c) are observed in the crystal structures and reproduced by gas-phase DFT calculations, without including the counter ions, which confirms that these geometrical effects are caused by the electronic structure, not by interactions with the alkali metal ions. The surprisingly good match between the solid-state and gas-phase geometries is seen by comparing the bond lengths (Fig. 3a and b) and from the overlayed projections in Fig. 4.

Taken together, the results presented here strongly support the conclusion that the 1^2−^ di-anion is globally aromatic. The diatropic ring current deduced from NMR spectroscopy is not just a magnetic effect; it reports on the global aromatic delocalization, and that delocalization is also expressed through the molecular geometry. The situation with the 1^4−^ tetra-anion is more complicated: the molecular structure indicates significant localization, yet the observation of a paratropic ring current indicates that there is some degree of global anti-aromaticity in this 28π system. Paradoxically, the observation of distortion and charge-localization in 1^4−^ (which must mitigate against anti-aromaticity) provides strong evidence for anti-aromaticity—it is difficult to identify other possible causes for the observed localization. The results from this work are relevant to understanding electronic delocalization in large π-conjugated macrocycles, and further insights into the structures of alkali metals salts of 1 may be useful for the development of materials for batteries.^5^

## Data availability

The data that support the findings of this study are available in the ESI† of this article. Crystallographic data have been deposited at the Cambridge crystallographic data. Deposition numbers: 2272266 (Li_2_-1^2−^), 2272267 (Na_2_-6THF-1^2−^), 2272268 (Na_2_-7THF-1^2−^), 2272269 (Na_2_-crown-1^2−^), 2272270 (K_2_-crypt-1^2−^) and 2272271 (Li_4_-1^4−^). These data can be obtained free of charge *via*https://www.ccdc.cam.ac.uk/data_request/cif, or by emailing data_request@ccdc.cam.ac.uk. Cartesian coordinates of calculated molecular geometries are available from the Zenodo public repository (https://zenodo.org/record/8239309 and https://doi.org/10.5281/zenodo.8239309).

## Author contributions

H. L. A. initiated the project. W. S. synthesized the neutral macrocycle 1 in Oxford and performed reduction and crystallization experiments under guidance of Y. Z. in Albany. Compounds were characterized by NMR and UV-vis-NIR spectroscopy by W. S. and Y. Z. Glassblowing was done by Y. Z. X-ray diffraction experiments and refinement were performed by Z. W. DFT calculations were done by W. S. The draft manuscript was written by W. S., and edited by H. L. A. and M. A. P. All authors revised the manuscript. Funding was secured by H. L. A. and M. A. P., and they supervised the whole project.

## Conflicts of interest

There are no conflicts to declare.

## Supplementary Material

SC-014-D3SC04251K-s001

SC-014-D3SC04251K-s002
