# Supplementary material for: Crystallographic evidence for global aromaticity in the di-anion and tetra-anion of a cyclophane hydrocarbon
Source: Chem Sci. 2023 Sep 20;14(48):14109–14. doi: 10.1039/d3sc04251k (PMC10718070; doi:10.1039/d3sc04251k)
Supplement: SC-014-D3SC04251K-s001 [file SC-014-D3SC04251K-s001.pdf]

Electronic Supplementary Material (ESI) for Chemical Science.
This journal is © The Royal Society of Chemistry 2023

Electronic Supporting Information

Crystallographic evidence for global aromaticity in the di-anion and tetra-anion of a cyclophane hydrocarbon

Wojciech Stawski,[a,b] Yikun Zhu,[b] Zheng Wei,[b] Marina A. Petrukhina,*[b] and Harry L. Anderson*[a]

a Department of Chemistry, University at Albany, State University of New York
Albany, NY 12222 (USA)
E-mail: mpetrukhina@albany.edu

b Department of Chemistry, University of Oxford
Chemistry Research Laboratory, Oxford OX1 3TA (UK)
E-mail: harry.anderson@chem.ox.ac.uk

Table of Contents

S1.	General Methods	S2
S2.	Synthetic Procedures	S2
S3.	X-ray Crystallography	S4
S4.	UV-vis-NIR Spectroscopy	S23
S5.	NMR Spectroscopy	S25
S6.	Theoretical Calculations	S34
S7.	HOMA Calculations	S39
S8.	References	S40

1. General Methods

Tetrahydrofuran (THF) and hexanes were purchased as dry from Sigma Aldrich and then freshly distilled over sodium with benzophenone as indicator before use. THF-d_8 for NMR purposes was purchased from Sigma Aldrich and distilled over sodium and potassium alloy in the presence of benzophenone under reduced pressure and then stored inside a glovebox. Alkali metals were purchased from Sigma Aldrich, washed with hexanes to remove mineral oil and stored inside a glovebox under argon. All reactions and follow-up procedures were performed in custom-made glass systems under an atmosphere of argon, using the 'break-and-seal' technique.[1] ^{1}H and ^{7}Li NMR spectra were recorded on Bruker Ascend 500 MHz NMR spectrometer. ^{1}H NMR spectra were referenced against the residual solvent peak (THF-d_8 δ_H = 3.58 ppm); for ^{7}Li NMR, 0.1 M LiCl in THF-d_8 (δ_{Li} = 0.00 ppm) was used as a reference. Samples were prepared in the NMR tubes inside a glovebox, wrapped with Parafilm and then quickly flame-sealed after removing from glovebox. UV-vis-NIR absorption measurements were carried out in a 1 cm path length quartz cuvette with PTFE cap at 298 K using Jasco V770 spectrophotometer. THF for UV-vis-NIR measurements was stored over lithium metal to remove residual oxygen/moisture. Samples were prepared inside a glovebox, the cuvette was closed tightly with a PTFE cap, wrapped with Parafilm, and removed from the glovebox. Semipreparative GPC was carried out on a Shimadzu recycling GPC system equipped with a LC-20 AD pump, SPD20A UV detector and a set of JAIGEL 3H (20 × 600 mm) and JAIGEL 4H (20 × 600 mm) columns in THF as the eluent at a flow rate of 3.5 mL/min.

2. Synthetic Procedures

[2$_4$]Paracyclophanetetraene was synthesized as described in the literature.[2] We found that during separation using GPC, parts of the tubing that were exposed to laboratory light got permanently yellow-colored. It is therefore recommended to wrap up visible parts of tubing with aluminum foil or perform the separation in the dark.

2.1. $[Li^+(THF)_4][\{Li^+(THF)_3\}C_{32}H_{24}^{2-}]$ (**Li$_2$-1^{2-}**)

THF (1.0 mL) was added to a customized glass system (Fig. S1, left) containing excess Li metal (6.0 mg, 0.86 mmol, 171 equiv.) and PCT (**1**, 2.0 mg, 0.005 mmol). The mixture was stirred at 25 °C under argon for 2 hours. The initial yellow color (neutral ligand) changed to orange in 20 min. The mixture was filtered after 2 hours while still orange (Fig. S1, right), and the orange filtrate was layered with anhydrous hexanes (3.0 mL). The ampule was sealed under argon and stored at 5 °C. Dark brown block-like crystals were present after 7 days. Yield: 3.5 mg, 70%. It is important to not keep the reaction for too long, otherwise the color changes to brown/red due to formation of tetra-reduced product.

^{1}H NMR (500 MHz, THF-d_8, −30 °C) δ 9.54 (s, 8H), 9.25 (s, 8H), –7.10 (s, 8H).

^{7}Li NMR (194 MHz, THF) δ –3.02.

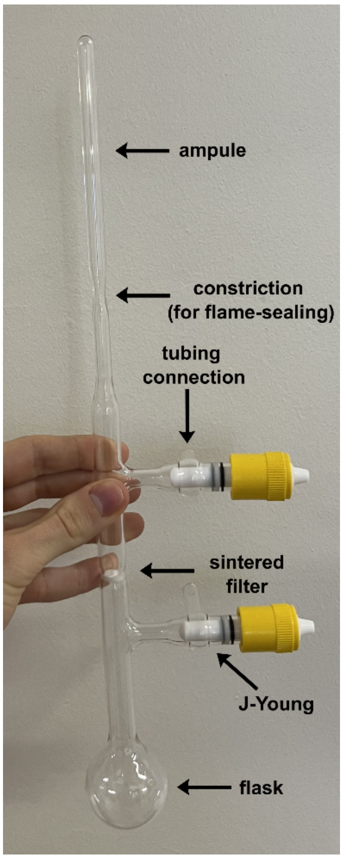

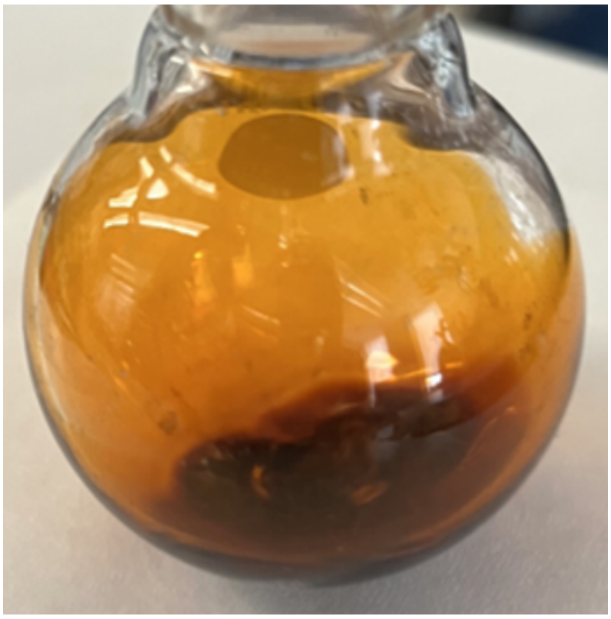

Figure S1. Left: Customized reaction vessel,[1] and right: Reaction mixture with Li at the dianion stage.

2.2. $[Na^+(THF)_3][Na^+(THF)_4][C_{32}H_{24}^{2-}]$ (**Na_2-7THF-1^{2-}**) and $[Na^+(THF)_3][Na^+(THF)_3][C_{32}H_{24}^{2-}]$ (**Na_2-6THF-1^{2-}**)

THF (1.0 mL) was added to a customized glass system containing excess Na metal (6.0 mg, 0.26 mmol, 52 equiv.) and PCT (**1**, 2.0 mg, 0.005 mmol). The mixture was stirred at 25 °C under argon. The initial yellow color (neutral ligand) changed to orange in 5 min. The mixture was filtered after another 20 minutes when the solution became intense orange, and the filtrate was layered with anhydrous hexanes (2.0 mL). The ampule was sealed under argon and stored at 5 °C. Brownish long plates with blue shine started forming after 30 min, crystallizing fully from the solution after 4 days. Diffraction experiments indicated formation of two types of solvates with different numbers of coordinated THF molecules. Yield: 4.5 mg, 95% calculated for **Na_2-7THF-1^{2-}**.
^{1}H NMR (500 MHz, THF, −30 °C) δ 9.57 (s, 8H), 9.27 (s, 8H), –6.94 (s, 8H).

2.3. $[Na^+(18\text{-crown-6})(THF)_2]_2[C_{32}H_{24}^{2-}]$ (**Na_2-crown-1^{2-}**)

THF (1.0 mL) was added to a customized glass system containing excess Na metal (6.0 mg, 0.26 mmol, 52 equiv.) PCT (**1**, 2.0 mg, 0.005 mmol) and 18-crown-6 (2.72 mg, 2.1 equiv., 0.01 mmol). The mixture was stirred at 25 °C under argon. The initial yellow color (neutral ligand) changed to orange in 20 min. The mixture was filtered after another 40 minutes when the solution became intense orange, and the filtrate was layered with anhydrous hexanes (2.0 mL). The ampule was sealed under argon and stored at 5 °C. Crystals started forming quickly and the layers were fully mixed after 5 days. Yield: 5.9 mg, 90%.
^{1}H NMR (500 MHz, THF-*d$_8$*, −90 °C) δ 9.49 (br. s, 8H), 9.19 (br. s, 8H), –7.05 (br. s, 8H).

2.4. $[K^+(cryptand)]_2[C_{32}H_{24}^{2-}]\cdot 1.3THF\cdot 0.3C_6H_{14}$ (**K_2-crypt-1^{2-}**)

THF (1.0 mL) was added to a customized glass system containing excess K metal (6.0 mg, 0.15 mmol, 31 equiv.) PCT (**1**, 2.0 mg, 0.005 mmol) and [2.2.2]cryptand (3.7 mg, 2.1 equiv., 0.10 mmol). The mixture was stirred at 25 °C under argon for 1.5 hour. The initial yellow color (neutral ligand) changed to orange in 10 minutes. The mixture was filtered after 1.5 hours, and the orange filtrate was layered with anhydrous hexanes (2.0 mL). The ampule was sealed under argon and stored at 5 °C. Dark brown blocks started appearing after one hour and they were fully precipitated after 3 days. Yield: 5.7 mg, 85%.
^{1}H NMR (500 MHz, THF, −80 °C) δ 9.46 (br. s, 8H), 9.23 (br. s, 8H), –6.76 (br. s, 8H).

2.5. $[Li^+(THF)_2]_4[C_{32}H_{24}^{4-}]$ (**Li_4-1^{4-}**)

THF (1.0 mL) was added to a customized glass system containing excess Li metal (6.0 mg, 0.86 mmol) and PCT (**1**, 2.0 mg, 0.005 mmol). The mixture was stirred at 25 °C under argon for 5 hours. The initial yellow color (neutral ligand) has changed to orange in 1 hour and to reddish brown in 4 hours (Fig. S2). The mixture was filtered after 5 hours, and the reddish-brown filtrate was layered with anhydrous hexanes (4.0 mL). The ampule was sealed under argon and stored at 5 °C. Dark brown blocks were present after 7 days. Yield: 1.5 mg, 30%. The yield is relatively low due to good solubility of the tetra-reduced product in the mixture of THF and hexanes.
^{1}H NMR (500 MHz, THF-*d$_8$*, 25 °C) δ 12.39 (s, 8H), 4.59 (s, 8H), 2.30 (s, 8H).
^{7}Li NMR (194 MHz, THF) δ 0.25 (very broad).

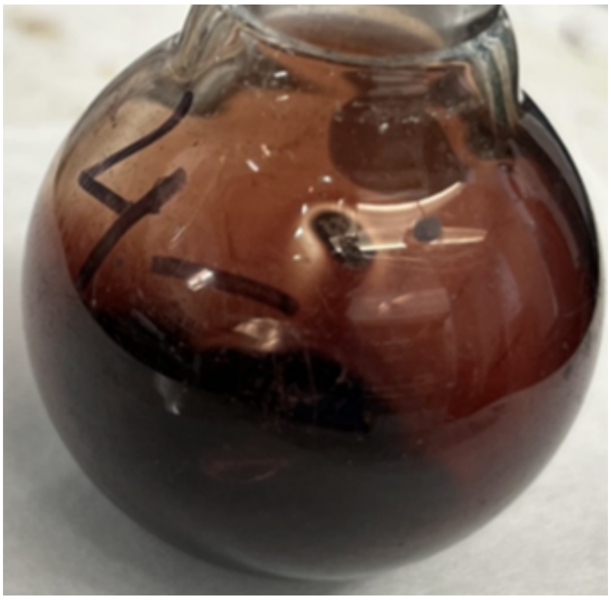

Figure S2. Reaction mixture with Li at the tetraanion stage.

3. X-ray crystallography

3.1. Measurement and refinement details

Data collection of **Li_2-1$^{2–}$**, **Na_2-7THF-1$^{2–}$**, **Na_2-6THF-1$^{2–}$**, **Na_2-crown-1$^{2–}$**, **K_2-crypt-1$^{2–}$**, and **Li_4-1$^{4–}$** were performed at 100(2) K on a Huber 4-circle system with a DECTRIS PILATUS3 X 2M(CdTe) pixel array detector using ϕ scans (synchrotron radiation at λ = 0.49594 Å) located at the Advanced Photon Source, Argonne National Laboratory (NSF's ChemMatCARS, Sector 15, Beamline 15-ID-D). The dataset reduction and integration were performed with the Bruker software package SAINT (version 8.38A).[3] Data were corrected for absorption effects using the empirical methods as implemented in SADABS (version 2016/2).[4] The structures were solved by SHELXT (version 2018/2)[5] and refined by full-matrix least-squares procedures using the Bruker SHELXTL (version 2019/2)[6] software package through the OLEX2 graphical interface.[7] All non-hydrogen atoms, including those in disordered parts, were refined anisotropically. Hydrogen atoms were included in idealized positions for structure factor calculations with U_{iso}(H) = 1.2 U_{eq}(C). In the structure model of **Li_2-1$^{2–}$**, five THF molecules were found to be disordered. In **Na_2-7THF-1$^{2–}$**, five THF molecules were found to be disordered. In **Na_2-6THF-1$^{2–}$**, two THF molecules were found to be disordered. In **Na_2-crown-1$^{2–}$**, the whole structure, except two sodium cations, was found to be disordered. In **K_2-crypt-1$^{2–}$**, one of the cryptand molecules, the THF molecule, the *n*-hexane molecule, and the **1$^{2–}$** were found to be disordered. In **Li_4-1$^{4–}$**, six THF molecules were found to be disordered. The disordered molecules were modeled with two orientations with their relative occupancies refined. The geometries of the disordered parts were restrained to be similar by using restraints SADI and DFIX. The anisotropic displacement parameters of the disordered molecules were restrained to have the same U_{ij} components by using the restraint SIMU, with a standard uncertainty of 0.01 Å^2. In each unit cell of **Na_2-crown-1$^{2–}$**, two THF solvent molecules were found to be severely disordered and removed by the Olex2's solvent mask subroutine.[7] The total void volume was 230.4 Å^3, equivalent to 6.31% of the unit cell's total volume. Further crystal and data collection details are listed in Table S1.

Table S1. Crystal data and structure refinement parameters for **Li_2-1$^{2–}$**, **Na_2-7THF-1$^{2–}$**, **Na_2-6THF-1$^{2–}$**, **Na_2-crown-1$^{2–}$**, **K_2-crypt-1$^{2–}$**, and **Li_4-1$^{4–}$**.

Compound	**Li_2-1$^{2–}$**	**Na_2-7THF-1$^{2–}$**	**Na_2-6THF-1$^{2–}$**
Empirical formula	$C_{60}H_{80}Li_2O_7$	$C_{60}H_{80}Na_2O_7$	$C_{56}H_{72}Na_2O_6$
Formula weight	927.12	959.22	887.11
Temperature (K)	100(2)	100(2)	100(2)
Wavelength (Å)	0.49594	0.49594	0.49594
Crystal system	Orthorhombic	Monoclinic	Monoclinic
Space group	$Pna2_1$	$P2_1/c$	$P2_1/c$
a (Å)	15.5976(13)	17.8448(5)	8.8122(3)
b (Å)	17.2486(14)	16.1337(4)	26.5491(8)
c (Å)	19.3869(16)	18.5018(5)	20.9892(7)
α (°)	90.00	90.00	90.00
β (°)	90.00	90.3280(10)	92.8960(10)
γ (°)	90.00	90.00	90.00
V (Å^3)	5215.8(7)	5326.6(2)	4904.3(3)
Z	4	4	4
ρ_{calcd} (g·cm^{-3})	1.181	1.196	1.201
μ (mm^{-1})	0.040	0.046	0.046
F(000)	2008	2072	1912
Crystal size (mm)	0.03 × 0.06 × 0.11	0.02 × 0.09 × 0.12	0.05 × 0.07 × 0.08
θ range for data collection (°)	1.103–24.864	1.109–31.281	1.267–24.924
Reflections collected	238473	359467	247080
Independent reflections	26236 [R_{int} = 0.0676][a]	48338 [R_{int} = 0.0539]	25085 [R_{int} = 0.0678]
Transmission factors (min/max)	0.4727/0.6718	0.6340/0.6751	0.5797/0.6984
Data/restraints/params.	26236/801/853	48338/885/821	25085/420/657
R_1,[b] wR_2[c] ($I > 2\sigma(I)$)	0.0502, 0.1397	0.0396, 0.1146	0.0575, 0.1456
R_1,[b] wR_2[c] (all data)	0.0581, 0.1425	0.0537, 0.1252	0.0809, 0.1609
Quality-of-fit[d]	1.081	1.038	1.018
CCDC number	2272266	2272268	2272267

[a] $R_{int} = \Sigma|F_o^2-<F_o^2>|/\Sigma|F_o^2|$. [b] $R_1 = \Sigma||F_o|-|F_c||/\Sigma|F_o|$. [c] $wR2 = [\Sigma[w(F_o^2-F_c^2)^2]/\Sigma[w(F_o^2)^2]]$. [d] Quality-of-fit = $[\Sigma[w(F_o^2-F_c^2)^2]/(N_{obs}-N_{params})]^{½}$, based on all data.

Table S1 (cont.). Crystal data and structure refinement parameters for **Li_2-1^{2-}**, **Na_2-7THF-1^{2-}**, **Na_2-6THF-1^{2-}**, **Na_2-crown-1^{2-}**, **K_2-crypt-1^{2-}**, and **Li_4-1^{4-}**.

Compound	**Na_2-crown-1^{2-}**	**K_2-crypt-1^{2-}**	**Li_4-1^{4-}**
Empirical formula	$C_{76}H_{112}Na_2O_{17}$	$C_{75.31}H_{111.31}K_2N_4O_{13.31}$	$C_{64}H_{88}Li_4O_8$
Formula weight	1343.63	1363.82	1013.10
Temperature (K)	100(2)	100(2)	100(2)
Wavelength (Å)	0.49594	0.49594	0.49594
Crystal system	Triclinic	Monoclinic	Triclinic
Space group	*P*-1	$P2_1/c$	*P*-1
a (Å)	12.0529(8)	21.9901(8)	12.0087(15)
b (Å)	15.8788(11)	18.2207(6)	12.8316(16)
c (Å)	20.3203(14)	20.0657(7)	19.699(3)
α (°)	87.647(2)	90.00	89.457(2)
β (°)	76.9210(10)	114.1120(10)	89.6290(10)
γ (°)	74.7180(10)	90.00	76.211(2)
V (Å^3)	3653.5(4)	7338.3(4)	2947.7(6)
Z	2	4	2
ρ_{calcd} (g·cm^{-3})	1.221	1.234	1.141
μ (mm^{-1})	0.047	0.082	0.039
F(000)	1452	2942.5	1096
Crystal size (mm)	0.03 × 0.04 × 0.06	0.06 × 0.08 × 0.12	0.06 × 0.09 × 0.12
θ range for data collection (°)	0.928–18.815	1.053–22.861	1.140–19.348
Reflections collected	105993	322345	147996
Independent reflections	16008 [R_{int} = 0.0486][a]	29401 [R_{int} = 0.0639][a]	14678 [R_{int} = 0.0950][a]
Transmission factors (min/max)	0.6568/0.7443	0.6185/0.7108	0.7168/0.7443
Data/restraints/params.	16008/3049/1610	29401/2154/1464	14678/711/881
R_1,[b] wR_2[c] ($I > 2\sigma(I)$)	0.0875, 0.2529	0.0471, 0.1339	0.0889, 0.2372
R_1,[b] wR_2[c] (all data)	0.1146, 0.2846	0.0619, 0.1426	0.1205, 0.2628
Quality-of-fit[d]	1.040	1.063	1.047
CCDC number	2272269	2272270	2272271

[a] $R_{int} = \Sigma|F_o^2-\langle F_o^2\rangle|/\Sigma|F_o^2|$. [b] $R_1 = \Sigma||F_o|-|F_c||/\Sigma|F_o|$. [c] $wR2 = [\Sigma[w(F_o^2-F_c^2)^2]/\Sigma[w(F_o^2)^2]]$. [d] Quality-of-fit = $[\Sigma[w(F_o^2-F_c^2)^2]/(N_{obs}-N_{params})]^{½}$, based on all data.

3.2. Crystal structures and structural parameters

Bond lengths, torsion and dihedral angles of neutral **1** are as in previously reported crystal structure.[8] We used these values rather than those from more recent work as in the latter an *AFIX 66* command was used during refinement on all phenyl rings which made all of them regular hexagons with bond length of 1.39 Å.[2] Another polymorph described in the more recent study features eight independent molecules of **1** and this dataset has low completeness, therefore we decided to not use it as a reference point during our comparisons.

3.2.1. $[Li^+(THF)_4][\{Li^+(THF)_3\}C_{32}H_{24}^{2-}]$ (**Li_2-1^{2-}**)

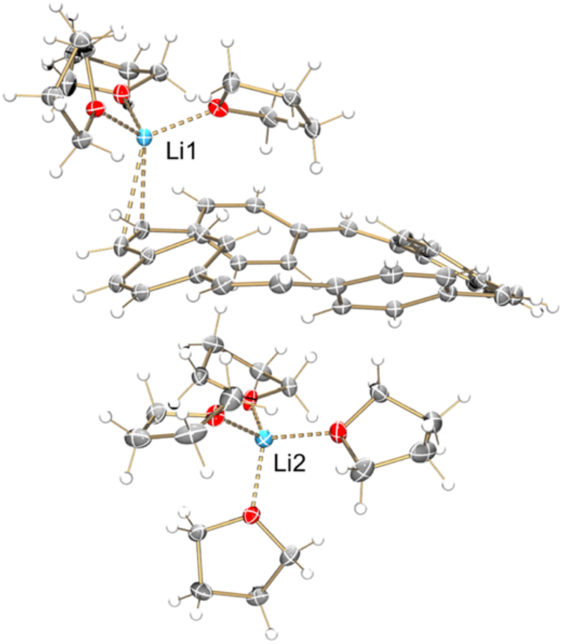

Figure S3. ORTEP drawing of the asymmetric unit of **Li_2-1^{2-}**, drawn with thermal ellipsoids at the 40% probability level. Color key: C gray, H white, O red, and Li sky-blue.

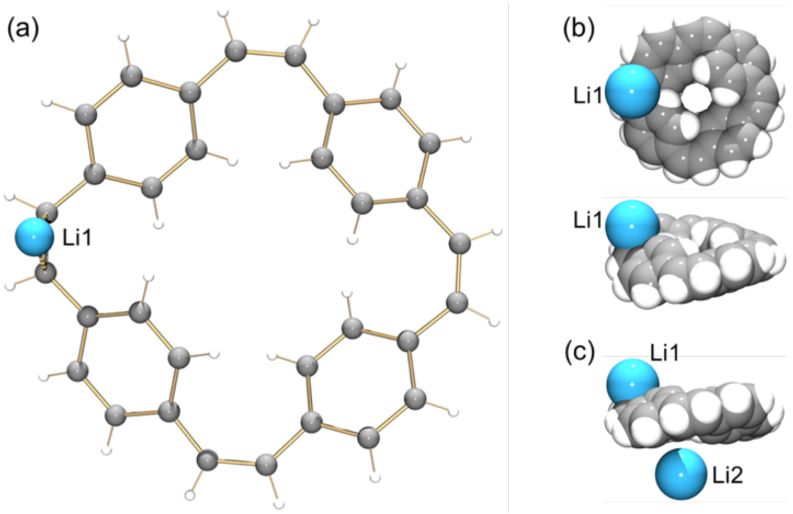

Figure S4. Li1 binding in **Li_2-1^{2-}**, (a) ball-and-stick model; (b) space-filling model. (c) Relative position for Li-ions and **1**$^{2-}$, space-filling model.

Table S2. Selected bond distances (Å) in **1** and **Li$_2$-1$^{2–}$** along with a labelling scheme. The Li cation binds to the ethylene bridge at the (C31/C32) site.

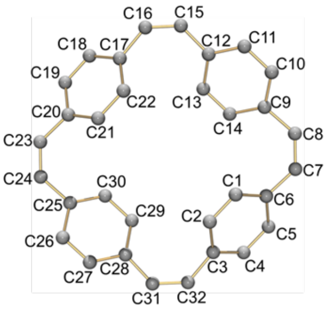

	1[8]	Li$_2$-1$^{2–}$		1[8]	Li$_2$-1$^{2–}$
C1–C2	1.373(5)	1.376(2)	C25–C26	1.407(5)	1.428(2)
C1–C6	1.401(5)	1.426(2)	C25–C30	1.408(5)	1.429(2)
C2–C3	1.403(5)	1.427(2)	C26–C27	1.362(5)	1.371(2)
C3–C4	1.390(5)	1.426(2)	C27–C28	1.424(5)	1.429(2)
C4–C5	1.347(5)	1.374(2)	C28–C29	1.384(5)	1.430(2)
C5–C6	1.393(5)	1.420(2)	C29–C30	1.354(5)	1.372(2)
C9–C10	1.391(5)	1.421(2)	C6–C7	1.471(5)	1.430(2)
C9–C14	1.399(5)	1.425(2)	C7–C8	1.306(5)	1.386(2)
C10–C11	1.365(5)	1.382(2)	C8–C9	1.475(5)	1.427(2)
C11–C12	1.396(5)	1.418(2)	C12–C15	1.478(5)	1.438(2)
C12–C13	1.404(5)	1.420(2)	C15–C16	1.310(5)	1.374(2)
C13–C14	1.370(5)	1.373(2)	C16–C17	1.462(5)	1.441(2)
C17–C18	1.400(5)	1.419(2)	C20–C23	1.485(5)	1.435(2)
C17–C22	1.410(5)	1.419(2)	C23–C24	1.317(5)	1.383(2)
C18–C19	1.364(5)	1.380(2)	C24–C25	1.467(5)	1.423(2)
C19–C20	1.381(5)	1.416(2)	C28–C31	1.454(5)	1.428(2)
C20–C21	1.407(5)	1.424(2)	C31–C32	1.313(5)	1.402(2)
C21–C22	1.365(5)	1.380(2)	C3–C32	1.464(5)	1.421(2)

* The C–C bond distances in **Li$_2$-1$^{2–}$**, which are significantly longer/shorter than the values in neutral **1**, are highlighted in blue/red color.

Table S3. Selected dihedral and torsion angles (°) in **1** and **Li_2-$1^{2–}$** along with a labelling scheme. The Li cation binds to the ethylene bridge at the (C31/C32) site.

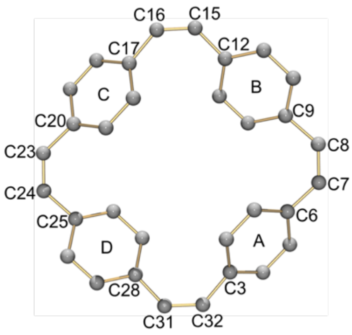

	**1**[8]	**Li_2-$1^{2–}$**		**1**[8]	**Li_2-$1^{2–}$**
A/B	44.2(3)	40.9(2)	C6–C7–C8–C9	–4.4(3)	–19.8(2)
A/C	121.8(3)	123.4(2)	C12–C15–C16–C17	3.7(3)	14.7(2)
A/D	51.2(3)	38.8(2)	C20–C23–C24–C25	–5.3(3)	–19.8(2)
B/C	43.5(3)	37.6(2)	C28–C31–C32–C3	8.6(3)	20.9(2)
B/D	104.6(3)	124.4(2)	C3–C6–C17–C20	5.8(3)	3.0(2)
C/D	46.4(3)	38.1(2)	C9–C12–C25–C28	–4.2(3)	–4.0(2)

In the solid-state structure, the C–H⋯*π* interactions (2.622(13)–2.924(13) Å) between the coordinated THF molecules and dianions contribute to the formation of an extended 3D network structure (Figure S5).

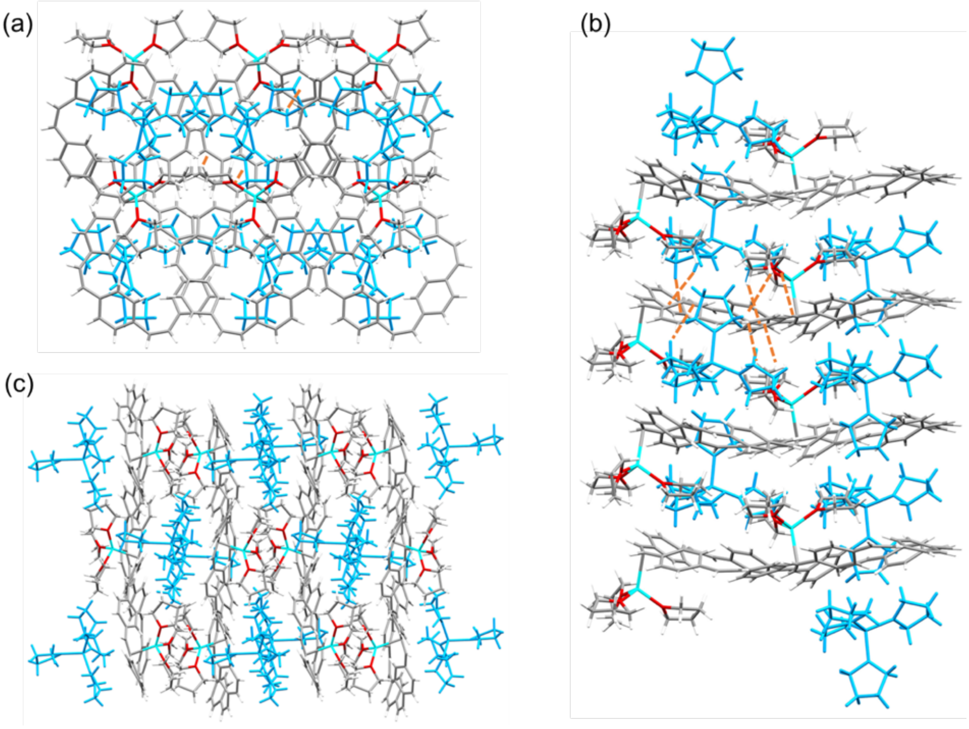

Figure S5. 3D network structure of **Li_2-$1^{2–}$** along the *a*, *b* and *c* axis, capped-stick model. C–H⋯*π* interactions (2.622(13)–2.924(13) Å) between two molecules are shown in orange. The solvent-separated Li^+ species are shown in different shades of blue.

3.2.2. $[Na^+(THF)_3][Na^+(THF)_4][C_{32}H_{24}{}^{2-}]$ (**Na_2-7THF-1^{2-}**) and $[Na^+(THF)_3][Na^+(THF)_3][C_{32}H_{24}{}^{2-}]$ (**Na_2-6THF-1^{2-}**)

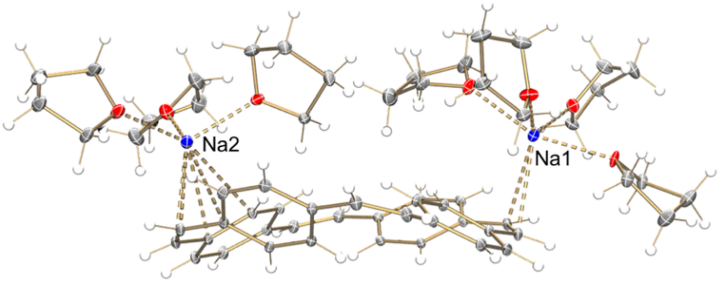

Figure S6. ORTEP drawing of the asymmetric unit of **Na_2-7THF-1^{2-}**, drawn with thermal ellipsoids at the 40% probability level. Color key: C gray, H white, O red, and Na blue.

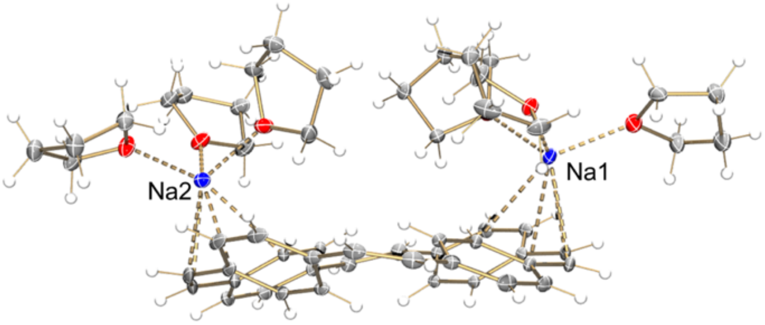

Figure S7. ORTEP drawing of the asymmetric unit of **Na_2-6THF-1^{2-}**, drawn with thermal ellipsoids at the 40% probability level. Color key: C gray, H white, O red, and Na blue.

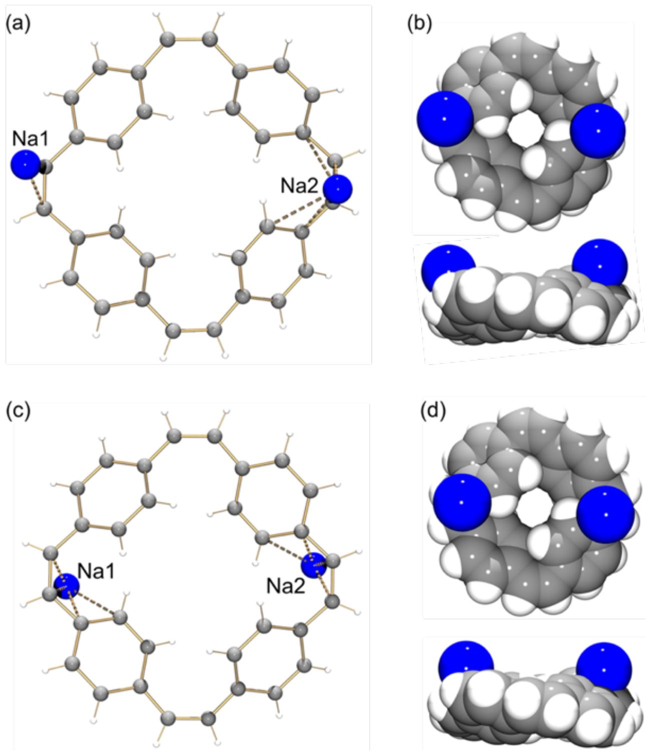

Figure S8. Na metal binding in **Na_2-7THF-1^{2-}** (top) and **Na_2-6THF-1^{2-}** (bottom), ball-and-stick model and space-filling model from two views.

Table S4. Selected bond distances (Å) in **1** and **Na_2-6THF-$1^{2–}$** along with a labelling scheme. The Na cations bind to the ethylene bridge at the (C23/C24) and (C7/C8) sites.

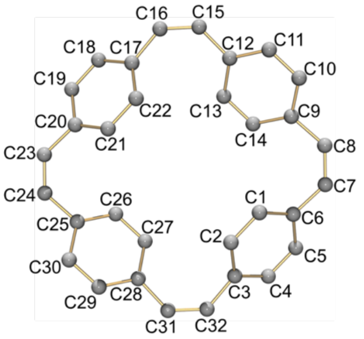

	1[8]	Na_2-6THF-$1^{2–}$		1[8]	Na_2-6THF-$1^{2–}$
C1–C2	1.373(5)	1.3758(11)	C25–C26	1.407(5)	1.4276(11)
C1–C6	1.401(5)	1.4293(11)	C25–C30	1.408(5)	1.4244(12)
C2–C3	1.403(5)	1.4234(12)	C26–C27	1.362(5)	1.4255(12)
C3–C4	1.390(5)	1.4242(13)	C27–C28	1.424(5)	1.3728(14)
C4–C5	1.347(5)	1.3723(15)	C28–C29	1.384(5)	1.3725(11)
C5–C6	1.393(5)	1.4279(14)	C29–C30	1.354(5)	1.4254(11)
C9–C10	1.391(5)	1.4259(14)	C6–C7	1.471(5)	1.4354(13)
C9–C14	1.399(5)	1.4283(11)	C7–C8	1.306(5)	1.3906(16)
C10–C11	1.365(5)	1.3722(18)	C8–C9	1.475(5)	1.4307(15)
C11–C12	1.396(5)	1.4249(13)	C12–C15	1.478(5)	1.4311(15)
C12–C13	1.404(5)	1.4252(12)	C15–C16	1.310(5)	1.3831(16)
C13–C14	1.370(5)	1.3707(13)	C16–C17	1.462(5)	1.4302(13)
C17–C18	1.400(5)	1.4236(14)	C20–C23	1.485(5)	1.4302(13)
C17–C22	1.410(5)	1.4251(12)	C23–C24	1.317(5)	1.3947(14)
C18–C19	1.364(5)	1.3725(15)	C24–C25	1.467(5)	1.4272(13)
C19–C20	1.381(5)	1.4268(13)	C28–C31	1.454(5)	1.4283(13)
C20–C21	1.407(5)	1.4301(11)	C31–C32	1.313(5)	1.3823(14)
C21–C22	1.365(5)	1.3733(11)	C3–C32	1.464(5)	1.4311(13)

* The C–C bond distances in **Na_2-6THF-$1^{2–}$**, which are significantly longer/shorter than the values in neutral **1**, are highlighted in blue/red color.

Table S5. Selected dihedral angles (°) in **1** and **Na$_2$-6THF-1^{2-}** along with a labelling scheme. The Na cations bind to the ethylene bridge at the (C23/C24) and (C7/C8) sites.

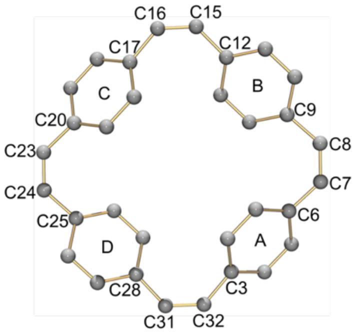

	**1**[8]	**Na$_2$-6THF-1^{2-}**		**1**[8]	**Na$_2$-6THF-1^{2-}**
A/B	44.2(3)	43.93(3)	C6–C7–C8–C9	–4.4(3)	–20.38(17)
A/C	121.8(3)	114.08(3)	C12–C15–C16–C17	3.7(3)	17.59(18)
A/D	51.2(3)	42.20(3)	C20–C23–C24–C25	–5.3(3)	–21.85(17)
B/C	43.5(3)	38.45(3)	C28–C31–C32–C3	8.6(3)	19.91(18)
B/D	104.6(3)	125.82(3)	C3–C6–C17–C20	5.8(3)	6.03(3)
C/D	46.4(3)	42.12(3)	C9–C12–C25–C28	–4.2(3)	–7.31(3)

Table S6. Selected bond distances (Å) in **1** and **Na$_2$-7THF-1$^{2–}$** along with a labelling scheme. The Na cations bind to the ethylene bridge at the (C23/C24) and (C7/C8) sites.

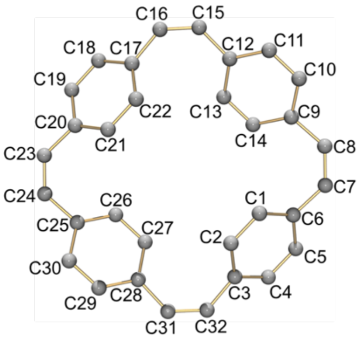

	**1**[8]	**Na$_2$-7THF-1$^{2–}$**		**1**[8]	**Na$_2$-7THF-1$^{2–}$**
C1–C2	1.373(5)	1.3770(5)	C25–C26	1.407(5)	1.4267(4)
C1–C6	1.401(5)	1.4300(4)	C25–C30	1.408(5)	1.4230(5)
C2–C3	1.403(5)	1.4240(4)	C26–C27	1.362(5)	1.3750(5)
C3–C4	1.390(5)	1.4248(5)	C27–C28	1.424(5)	1.4232(4)
C4–C5	1.347(5)	1.3784(5)	C28–C29	1.384(5)	1.4222(5)
C5–C6	1.393(5)	1.4258(5)	C29–C30	1.354(5)	1.3797(5)
C9–C10	1.391(5)	1.4266(5)	C6–C7	1.471(5)	1.4313(5)
C9–C14	1.399(5)	1.4285(4)	C7–C8	1.306(5)	1.3967(5)
C10–C11	1.365(5)	1.3800(5)	C8–C9	1.475(5)	1.4347(4)
C11–C12	1.396(5)	1.4246(5)	C12–C15	1.478(5)	1.4306(4)
C12–C13	1.404(5)	1.4286(4)	C15–C16	1.310(5)	1.3876(5)
C13–C14	1.370(5)	1.3761(4)	C16–C17	1.462(5)	1.4319(5)
C17–C18	1.400(5)	1.4246(5)	C20–C23	1.485(5)	1.4324(5)
C17–C22	1.410(5)	1.4258(4)	C23–C24	1.317(5)	1.3946(5)
C18–C19	1.364(5)	1.3806(5)	C24–C25	1.467(5)	1.4339(4)
C19–C20	1.381(5)	1.4260(4)	C28–C31	1.454(5)	1.4316(4)
C20–C21	1.407(5)	1.4294(4)	C31–C32	1.313(5)	1.3862(5)
C21–C22	1.365(5)	1.3763(4)	C3–C32	1.464(5)	1.4327(5)

* The C–C bond distances in **Na$_2$-7THF-1$^{2–}$**, which are significantly longer/shorter than the values in neutral **1**, are highlighted in blue/red color.

Table S7. Selected dihedral and torsion angles (°) in **1** and **Na$_2$-7THF-1^{2-}** along with a labelling scheme. The Na cations bind to the ethylene bridge at the (C23/C24) and (C7/C8) sites.

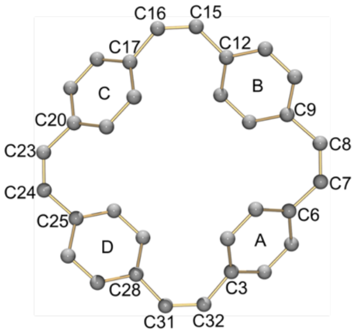

	**1**[8]	**Na$_2$-7THF-1^{2-}**		**1**[8]	**Na$_2$-7THF-1^{2-}**
A/B	44.2(3)	47.624(11)	C6–C7–C8–C9	–4.4(3)	–20.86(6)
A/C	121.8(3)	117.601(11)	C12–C15–C16–C17	3.7(3)	22.26(6)
A/D	51.2(3)	41.214(11)	C20–C23–C24–C25	–5.3(3)	–21.44(6)
B/C	43.5(3)	47.783(11)	C28–C31–C32–C3	8.6(3)	19.03(6)
B/D	104.6(3)	111.687(12)	C3–C6–C17–C20	5.8(3)	–10.344(16)
C/D	46.4(3)	43.453(11)	C9–C12–C25–C28	–4.2(3)	9.855(13)

In the solid-state structure of **Na$_2$-7THF-1^{2-}**, the C–H···π interactions (2.497(6)–2.941(6) Å) between the coordinated THF molecules and anions contribute to the formation of an extend 2D layer structure.

In the solid-state structure of **Na$_2$-6THF-1^{2-}**, the C–H···π interactions (2.594(6)–3.017(6) Å) between the coordinated THF molecules and anions contribute to the formation of a zig-zag 2D layer structure.

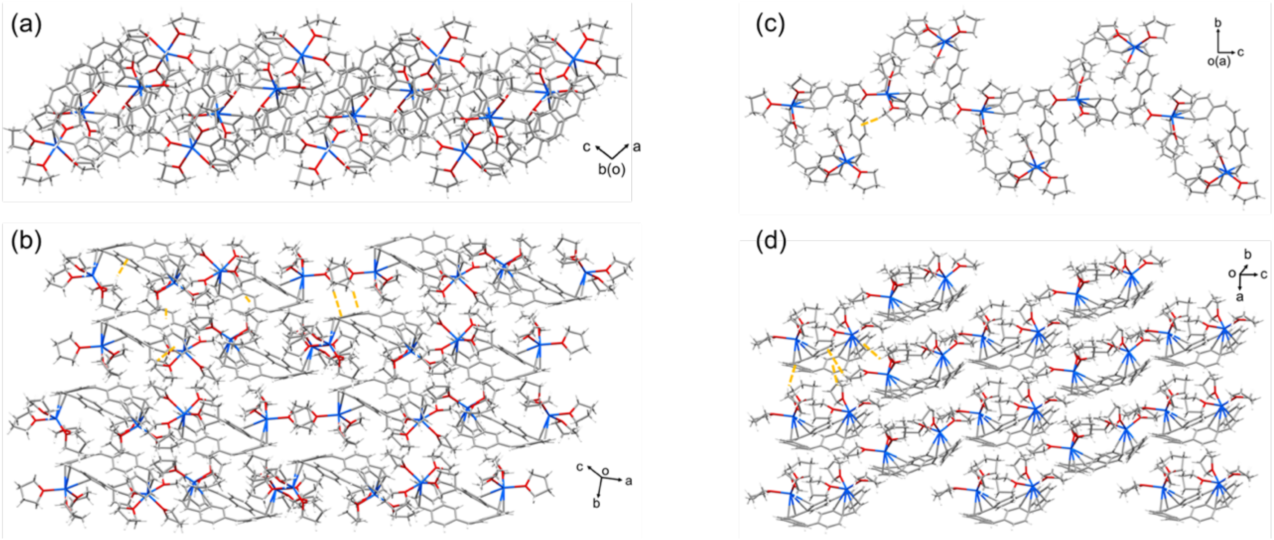

Figure S9. 2D layer structure of **Na$_2$-7THF-1^{2-}** (left) and **Na$_2$-6THF-1^{2-}** (right), capped-stick model. C–H···π interactions are shown in orange.

3.2.3. $[Na^+(C_{12}H_{24}O_6)(THF)_2]_2[C_{32}H_{24}^{2-}]$ (**Na_2-crown-1^{2-}**)

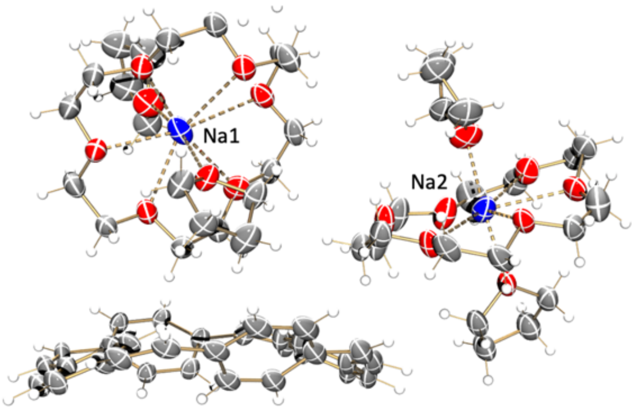

Figure S10. ORTEP drawing of the asymmetric unit of **Na_2-crown-1^{2-}**, drawn with thermal ellipsoids at the 40% probability level. Color key: C gray, H white, O red, and Na blue. Interstitial solvent molecules are omitted for clarity.

Table S8. Selected bond distances (Å) in **1** and **Na_2-crown-1^{2-}** along with a labelling scheme.

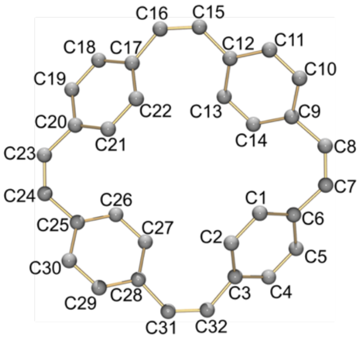

	**1**	**Na_2-crown-1^{2-}**		**1**	**Na_2-crown-1^{2-}**
C1–C2	1.373(5)	1.360(6)	C25–C26	1.407(5)	1.431(5)
C1–C6	1.401(5)	1.431(5)	C25–C30	1.408(5)	1.427(6)
C2–C3	1.403(5)	1.422(6)	C26–C27	1.362(5)	1.361(6)
C3–C4	1.390(5)	1.420(7)	C27–C28	1.424(5)	1.425(7)
C4–C5	1.347(5)	1.371(7)	C28–C29	1.384(5)	1.420(7)
C5–C6	1.393(5)	1.419(7)	C29–C30	1.354(5)	1.363(6)
C9–C10	1.391(5)	1.407(8)	C6–C7	1.471(5)	1.435(7)
C9–C14	1.399(5)	1.431(8)	C7–C8	1.306(5)	1.396(8)
C10–C11	1.365(5)	1.40(1)	C8–C9	1.475(5)	1.413(8)
C11–C12	1.396(5)	1.412(9)	C12–C15	1.478(5)	1.403(11)
C12–C13	1.404(5)	1.435(6)	C15–C16	1.310(5)	1.399(12)
C13–C14	1.370(5)	1.354(8)	C16–C17	1.462(5)	1.423(8)
C17–C18	1.400(5)	1.375(10)	C20–C23	1.485(5)	1.429(7)
C17–C22	1.410(5)	1.431(6)	C23–C24	1.317(5)	1.388(6)
C18–C19	1.364(5)	1.395(8)	C24–C25	1.467(5)	1.422(6)
C19–C20	1.381(5)	1.434(6)	C28–C31	1.454(5)	1.425(7)
C20–C21	1.407(5)	1.388(8)	C31–C32	1.313(5)	1.373(7)
C21–C22	1.365(5)	1.374(7)	C3–C32	1.464(5)	1.448(7)

Table S9. Selected dihedral and torsion angles (°) in **1** and **Na$_2$-crown-1^{2-}** along with a labelling scheme.

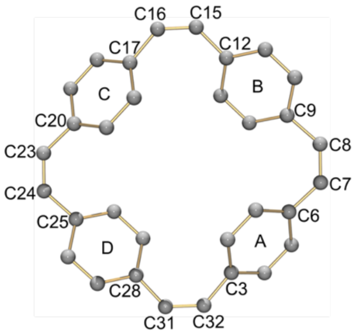

	**1**[8]	**Na$_2$-crown-1^{2-}**
A/B	44.2(3)	41.9(2)
A/C	121.8(3)	51.3(2)
A/D	51.2(3)	44.8(2)
B/C	43.5(3)	46.48(18)
B/D	104.6(3)	74.1(2)
C/D	46.4(3)	42.80(18)
C6–C7–C8–C9	–4.4(3)	-19.8(12)
C12–C15–C16–C17	3.7(3)	22.5(19)
C20–C23–C24–C25	–5.3(3)	-20.5(11)
C28–C31–C32–C3	8.6(3)	20.9(15)
C3–C6–C17–C20	5.8(3)	7.8(2)
C9–C12–C25–C28	–4.2(3)	-6.6(2)

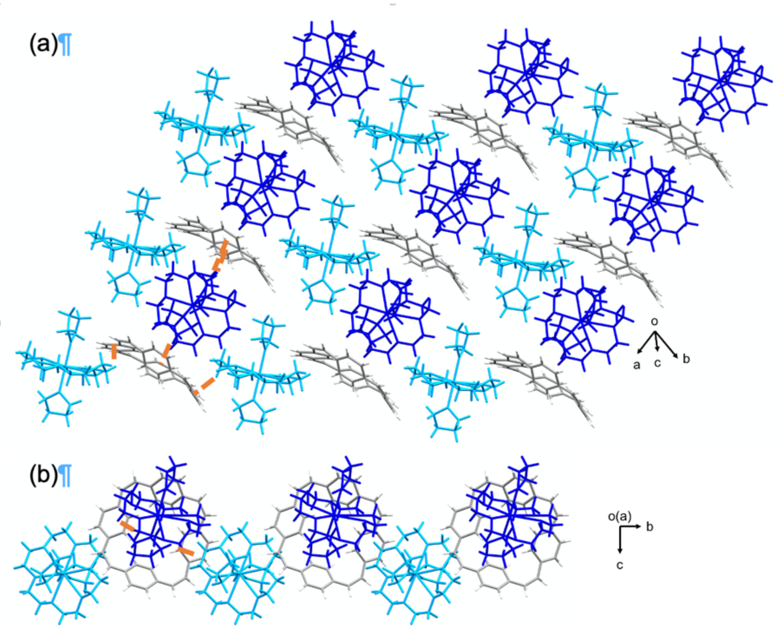

Figure S11. Solid-state packing of **Na$_2$-crown-1$^{2–}$** with C–H···π interactions (2.433(17)–2.943(17) Å.

3.2.4. $[K^+(C_{18}H_{36}N_2O_6)]_2[C_{32}H_{24}{}^{2–}]\cdot 1.3THF\cdot 0.3C_6H_{14}$ (**K$_2$-crypt-1$^{2–}$**)

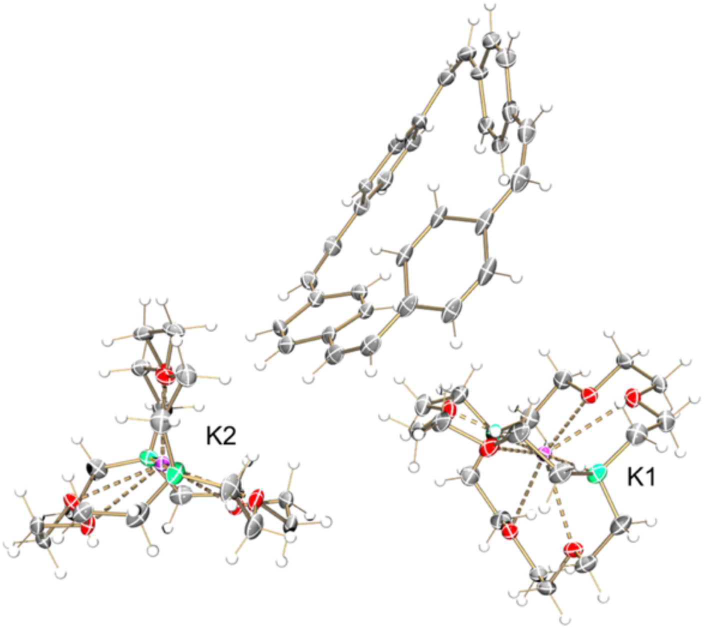

Figure S12. ORTEP drawing of the asymmetric unit of **K$_2$-crypt-1$^{2–}$**, drawn with thermal ellipsoids at the 40% probability level. Color key: C gray, H white, O red, N spring-green, and K dark-orchid. Interstitial solvent molecules are omitted for clarity.

Table S10. Selected bond distances (Å) in **1** and **K_2-crypt-1$^{2–}$** along with a labelling scheme.

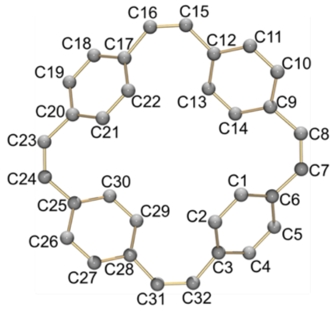

	**1**[8]	**K_2-crypt-1$^{2–}$**		**1**[8]	**K_2-crypt-1$^{2–}$**
C1–C2	1.373(5)	1.326(13)	C25–C26	1.407(5)	1.435(14)
C1–C6	1.401(5)	1.450(14)	C25–C30	1.408(5)	1.373(19)
C2–C3	1.403(5)	1.426(13)	C26–C27	1.362(5)	1.363(10)
C3–C4	1.390(5)	1.445(12)	C27–C28	1.424(5)	1.379(19)
C4–C5	1.347(5)	1.344(7)	C28–C29	1.384(5)	1.386(18)
C5–C6	1.393(5)	1.440(8)	C29–C30	1.354(5)	1.370(16)
C9–C10	1.391(5)	1.427(8)	C6–C7	1.471(5)	1.441(10)
C9–C14	1.399(5)	1.452(15)	C7–C8	1.306(5)	1.352(7)
C10–C11	1.365(5)	1.347(7)	C8–C9	1.475(5)	1.414(9)
C11–C12	1.396(5)	1.404(12)	C12–C15	1.478(5)	1.418(10)
C12–C13	1.404(5)	1.440(15)	C15–C16	1.310(5)	1.387(13)
C13–C14	1.370(5)	1.342(15)	C16–C17	1.462(5)	1.441(14)
C17–C18	1.400(5)	1.475(16)	C20–C23	1.485(5)	1.434(17)
C17–C22	1.410(5)	1.428(17)	C23–C24	1.317(5)	1.378(10)
C18–C19	1.364(5)	1.358(12)	C24–C25	1.467(5)	1.414(15)
C19–C20	1.381(5)	1.420(13)	C28–C31	1.454(5)	1.451(17)
C20–C21	1.407(5)	1.477(19)	C31–C32	1.313(5)	1.371(9)
C21–C22	1.365(5)	1.410(19)	C3–C32	1.464(5)	1.406(10)

* The C–C bond distances in **K_2-crypt-1$^{2–}$**, which are significantly longer/shorter than the values in neutral **1**, are highlighted in blue/red color.

Table S11. Selected dihedral and torsion angles (°) in **1** and **K_2-crypt-1$^{2–}$** along with a labelling scheme.

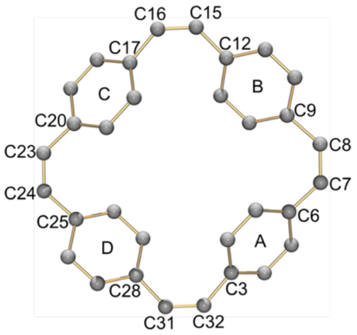

	**1**[8]	**K_2-crypt-1$^{2–}$**
A/B	44.2(3)	45.2(14)
A/C	121.8(3)	125.4(14)
A/D	51.2(3)	38.6(14)
B/C	43.5(3)	44.8(14)
B/D	104.6(3)	113.4(14)
C/D	46.4(3)	39.9(14)
C6–C7–C8–C9	–4.4(3)	–18.7(14)
C12–C15–C16–C17	3.7(3)	20.8(14)
C20–C23–C24–C25	–5.3(3)	–21.7(14)
C28–C31–C32–C3	8.6(3)	17.7(14)
C3–C6–C17–C20	5.8(3)	–8.9(14)
C9–C12–C25–C28	–4.2(3)	8.4(14)

In the solid-state structure, the C–H···π interactions (2.312(5)–2.904(5) Å) between the [2.2.2]cryptand molecules and anions contribute to the formation of an 1D column structure (Figure S13).

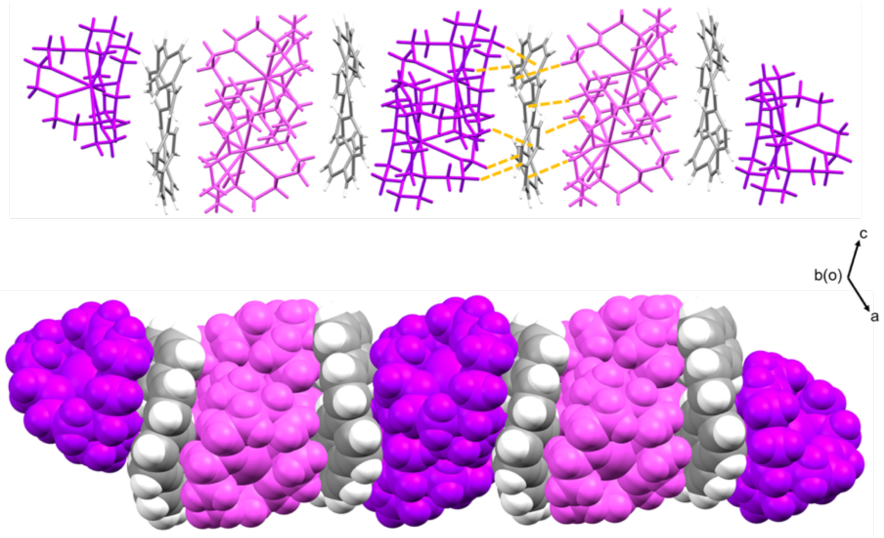

Figure S13. 1D column structure of **K_2-crypt-1$^{2–}$**, capped-stick model and space-filling model. C–H···π interactions (2.312(5)–2.904(5) Å) between two molecules are shown in yellow.

3.2.5. $[Li^{+}(THF)_2]_4[C_{32}H_{24}^{4-}]$ (**Li_4-1^{4-}**)

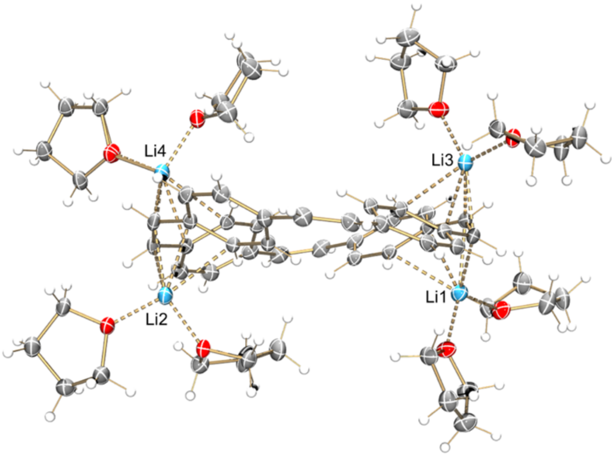

Figure S14. ORTEP drawing of the asymmetric unit of **Li_4-1^{4-}**, drawn with thermal ellipsoids at the 40% probability level. Color key: C gray, H white, O red, and Li sky-blue.

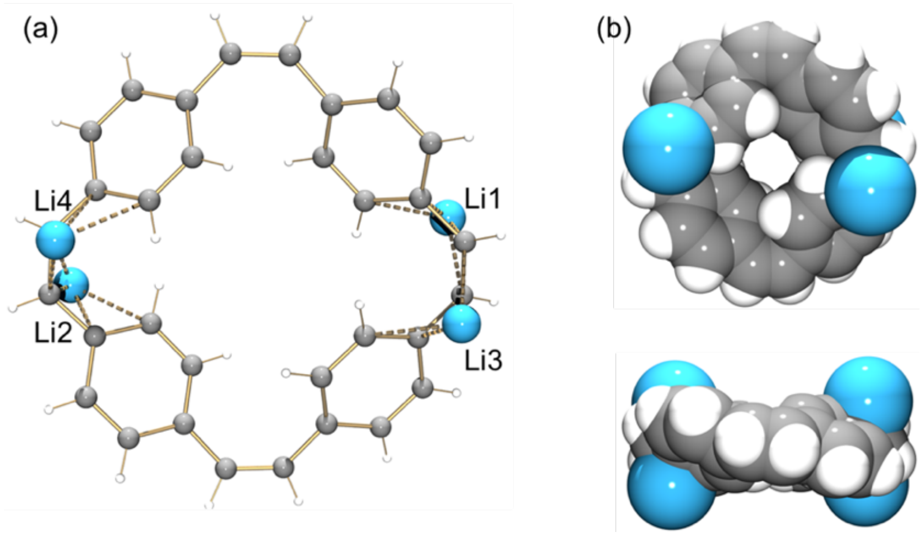

Figure S15. Li metal binding in **Li_4-1^{4-}**, (a) ball-and-stick model; (b) space-filling model from two views.

Table S12. Selected bond distances (Å) in **1** and **Li_4-1^{4-}** along with a labelling scheme. The Li cations bind to the ethylene bridge at the (C23/C24) and (C7/C8) sites.

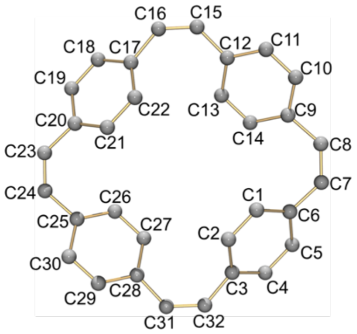

	**1**[8]	**Li_4-1^{4-}**		**1**[8]	**Li_4-1^{4-}**
C1–C2	1.373(5)	1.374(4)	C25–C26	1.407(5)	1.437(3)
C1–C6	1.401(5)	1.449(4)	C25–C30	1.408(5)	1.440(4)
C2–C3	1.403(5)	1.415(4)	C26–C27	1.362(5)	1.385(3)
C3–C4	1.390(5)	1.425(4)	C27–C28	1.424(5)	1.401(4)
C4–C5	1.347(5)	1.359(4)	C28–C29	1.384(5)	1.423(4)
C5–C6	1.393(5)	1.437(4)	C29–C30	1.354(5)	1.365(4)
C9–C10	1.391(5)	1.440(4)	C6–C7	1.471(5)	1.405(4)
C9–C14	1.399(5)	1.440(4)	C7–C8	1.306(5)	1.469(4)
C10–C11	1.365(5)	1.363(4)	C8–C9	1.475(5)	1.413(4)
C11–C12	1.396(5)	1.421(4)	C12–C15	1.478(5)	1.466(4)
C12–C13	1.404(5)	1.411(4)	C15–C16	1.310(5)	1.351(5)
C13–C14	1.370(5)	1.383(4)	C16–C17	1.462(5)	1.458(4)
C17–C18	1.400(5)	1.417(4)	C20–C23	1.485(5)	1.411(4)
C17–C22	1.410(5)	1.410(4)	C23–C24	1.317(5)	1.476(4)
C18–C19	1.364(5)	1.363(4)	C24–C25	1.467(5)	1.422(4)
C19–C20	1.381(5)	1.443(4)	C28–C31	1.454(5)	1.464(4)
C20–C21	1.407(5)	1.441(4)	C31–C32	1.313(5)	1.351(4)
C21–C22	1.365(5)	1.381(4)	C3–C32	1.464(5)	1.457(4)

* The C–C bond distances in **Li_4-1^{4-}**, which are significantly longer/shorter than the values in neutral **1**, are highlighted in blue/red color.

Table S13. Selected dihedral and torsion angles (°) in **1** and **Li_4-1^{4-}** along with a labelling scheme. The Li cations bind to the ethylene bridge at the (C23/C24) and (C7/C8) sites.

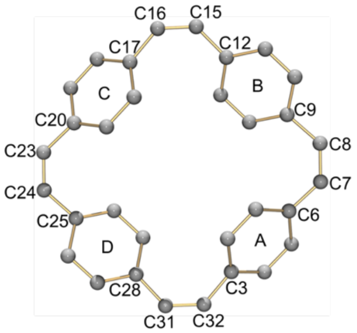

	**1**[8]	**Li-$_4$$1^{4-}$**		**1**[8]	**Li_4-1^{4-}**
A/B	44.2(3)	47.6(3)	C6–C7–C8–C9	–4.4(3)	–51.5(3)
A/C	121.8(3)	115.4(3)	C12–C15–C16–C17	3.7(3)	9.4(3)
A/D	51.2(3)	41.6(3)	C20–C23–C24–C25	–5.3(3)	–48.3(3)
B/C	43.5(3)	43.0(3)	C28–C31–C32–C3	8.6(3)	10.2(3)
B/D	104.6(3)	113.6(3)	C3–C6–C17–C20	5.8(3)	–0.4(3)
C/D	46.4(3)	47.5(3)	C9–C12–C25–C28	–4.2(3)	–7.1(3)

In the solid-state structure, the C–H···π interactions (2.613(6)–2.750(6) Å) between the coordinated THF molecules and anions contribute to the formation of an extended 3D network structure (Figure S16).

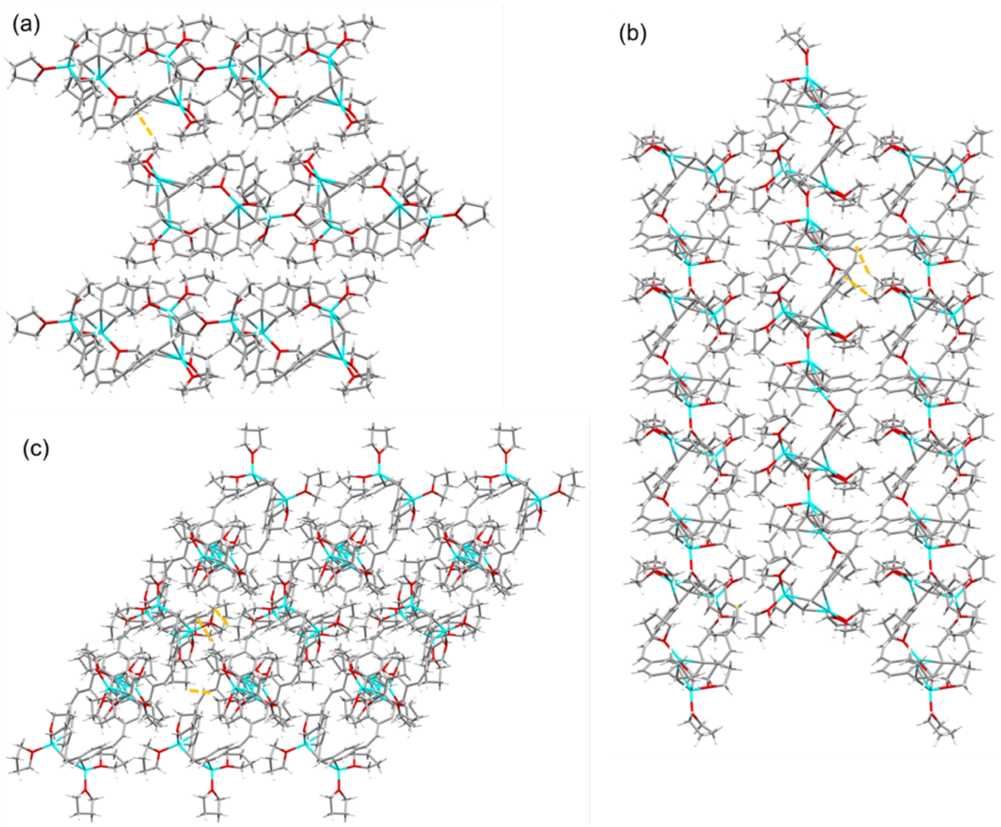

Figure S16. 3D network structure of **Li_4-1^{4-}** along the $\vec{a}$, $\vec{b}$ and $\vec{c}$ axis, capped-stick model. C–H···π interactions (2.613(6)–2.750(6) Å) between two molecules are shown in orange.

3.2.6. Overall comparison of bond lengths in the linkers

Table S14. Comparison of C-C bond lengths.

	Li_2-**1**$^{2-}$	Na_2-6THF-**1**$^{2-}$	Na_2-7THF-**1**$^{2-}$	K_2-crypt-**1**$^{2-}$	Na_2-crown-**1**$^{2-}$	**1**	Li_4-**1**$^{4-}$
**C=C cooridnated**	1.402(2)	1.3947(14)	1.3946(5)				1.476(4)
		1.3906(16)	1.3967(5)				1.469(4)
**C=C other**	1.383(2)	1.3831(16)	1.3862(5)	1.352(7)	1.399(12)	1.310(5)	1.351(4)
	1.386(2)	1.3823(14)	1.3876(5)	1.371(9)	1.396(8)	1.306(5)	1.351(5)
	1.374(2) opposite			1.378(10)	1.373(7)	1.313(5)	
				1.387(13)	1.388(6)	1.317(5)	
**C-C coord.**	1.421(2)	1.4354(13)	1.4313(5)				1.422(4)
	1.428(2)	1.4307(15)	1.4347(4)				1.411(4)
		1.4302(13)	1.4324(5)				1.413(4)
		1.4272(13)	1.4339(4)				1.405(4)
**C-C other**	1.441(2) opposite	1.4311(13)	1.4313(5)	1.418(10)	1.403(11)	1.471(5)	1.457(4)
	1.438(2) opposite	1.4283(13)	1.4347(4)	1.441(14)	1.423(8)	1.464(5)	1.464(4)
	1.427(2)	1.4311(15)	1.4324(5)	1.434(17)	1.413(8)	1.467(5)	1.466(4)
	1.430(2)	1.4302(13)	1.4339(4)	1.414(15)	1.435(7)	1.462(5)	1.458(4)
	1.423(2)			1.451(17)	1.448(7)	1.475(5)	
	1.435(2)			1.406(10)	1.425(7)	1.454(5)	
				1.441(10)	1.422(6)	1.485(5)	
				1.414(9)	1.429(7)	1.478(5)	

Table S15. Comparison of root mean square deviations (RMSD) from planes of the carbon atoms in the vinylene linkers, calculated using Olex2 software.[7]

	RMSD / Å
**1**[8]	0.138
Li_2-**1**$^{2-}$	0.144
Na_2-6THF-**1**$^{2-}$	0.212
Na_2-7THF-**1**$^{2-}$	0.299
K_2-crypt-**1**$^{2-}$	0.245
Na_2-crown-**1**$^{2-}$	0.235
Li_4-**1**$^{4-}$	0.235

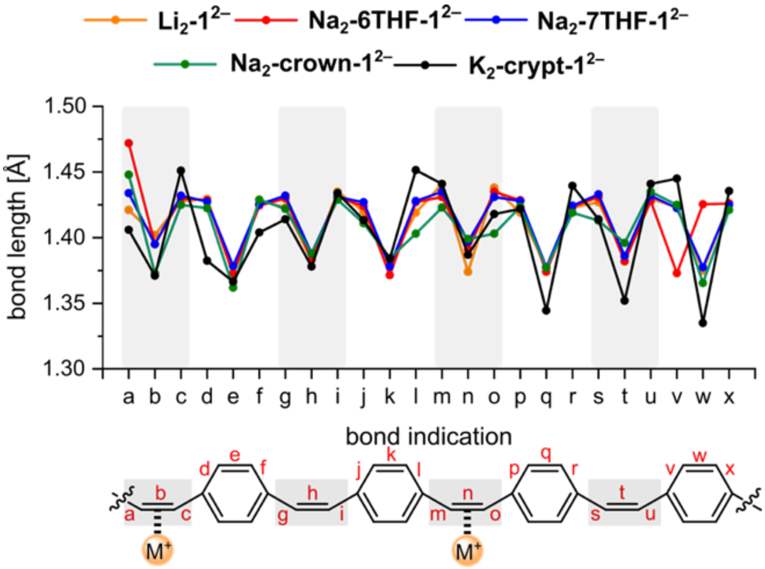

Figure S17. Comparison of crystallographic bond-length alternation in five dianion salts. Bonds in the vinylene linkers are marked in gray.

4. UV-vis-NIR spectroscopy

4.1 UV-vis-NIR monitoring of reaction of lithium metal with 1

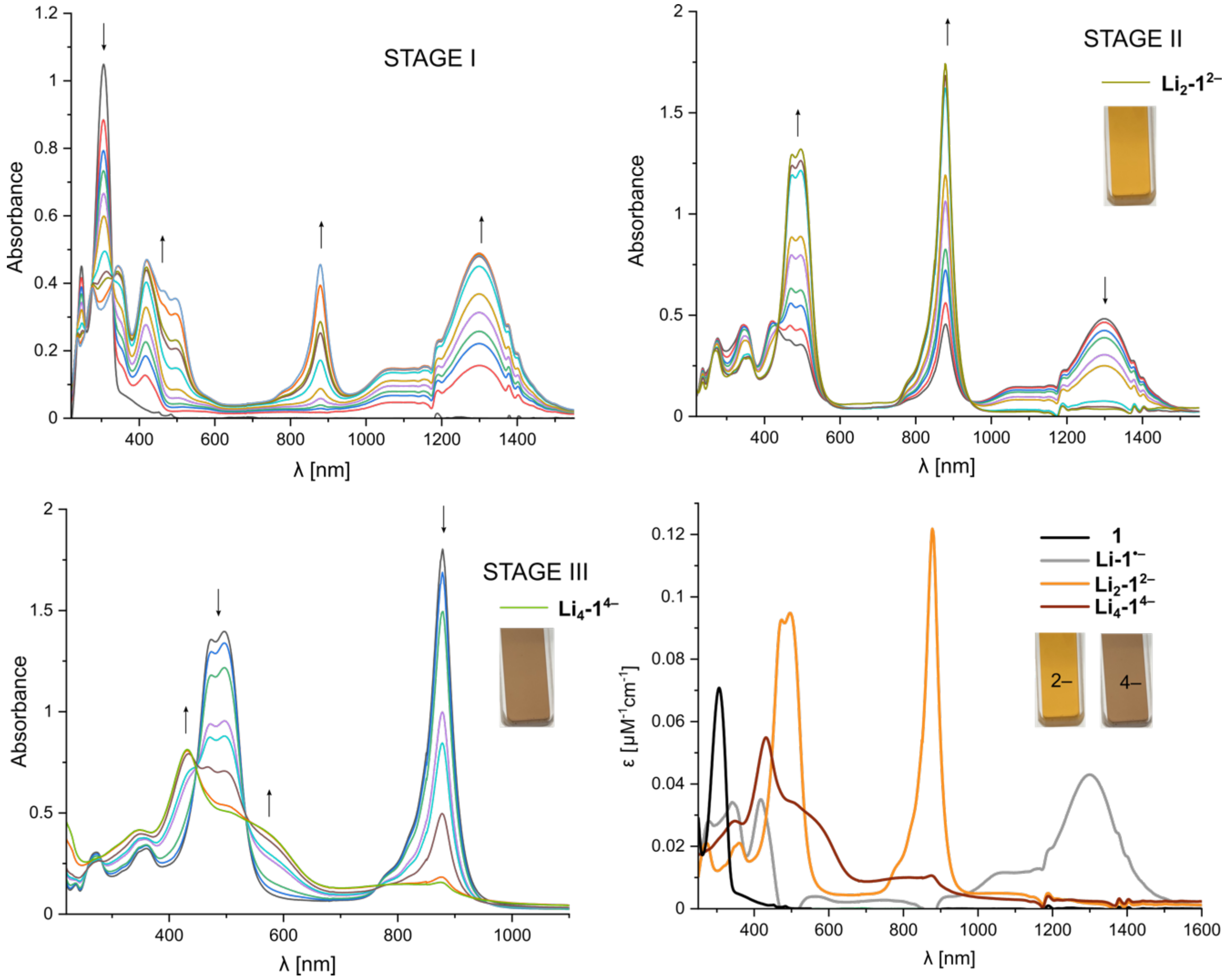

Figure S18. Progress of UV-vis-NIR monitored reaction of **1** with Li metal and spectra comparison with calculated molar absorption coefficients. The spectrum of the monoanion on the bottom-right picture was obtained by subtracting the spectrum of the dianion from a mixture of monoanion and dianion as it was not possible to record a spectrum of a pure monoanion separately. All spectra recorded in THF at 25 °C.

4.2 UV-vis-NIR spectra monitoring of reaction of sodium metal with 1

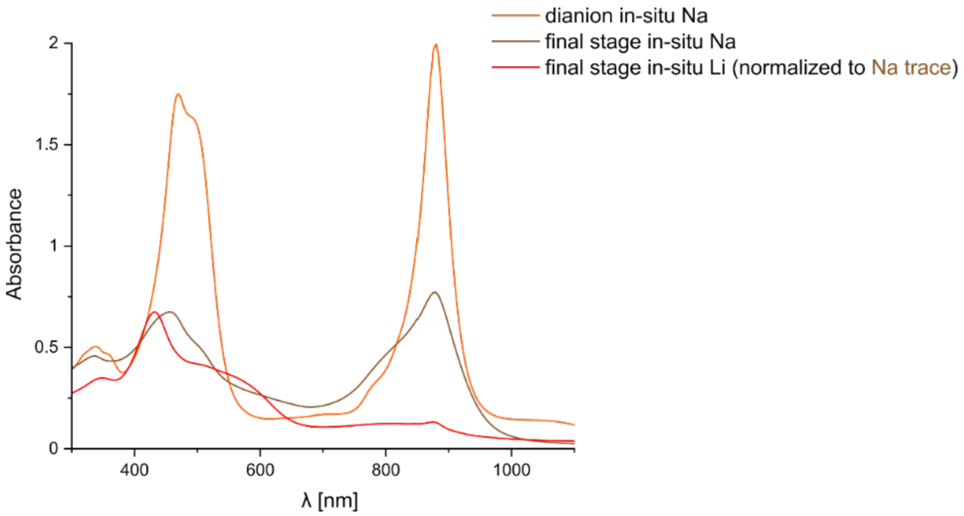

Figure S19.UV-vis-NIR spectra of dianion generated during in-situ reaction of **1** with sodium compared with the final stage of the reduction with Na and Li metals. All spectra recorded in THF at 25 °C.

4.3 UV-vis-NIR spectra monitoring of reaction of potassium metal with 1

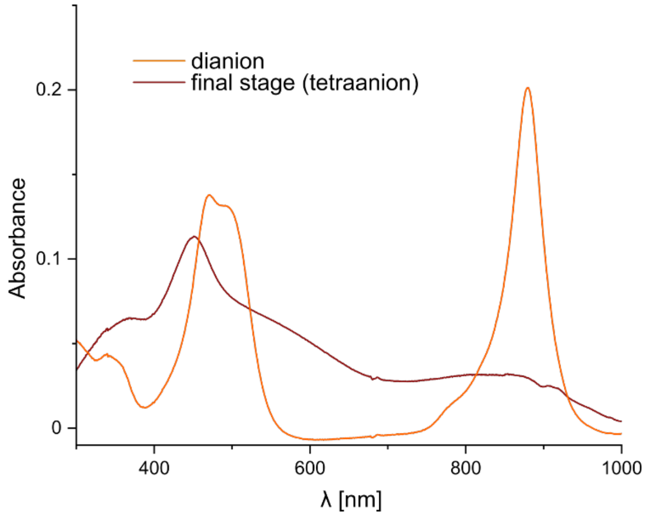

Figure S20. UV-vis-NIR spectra of potassium-generated dianion and final stage of reduction, indicating formation of the tetraanion (very low solubility). All spectra recorded in THF at 25 °C.

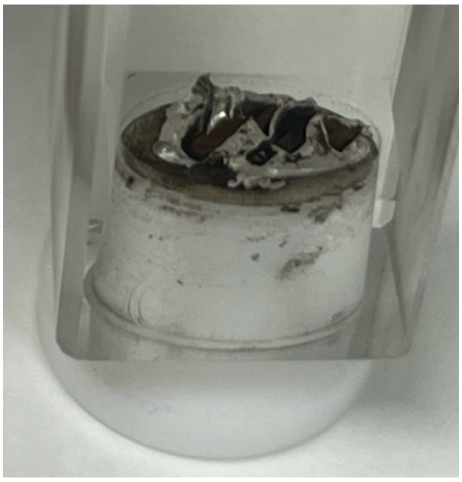

Figure S21. Black surface of potassium indicating precipitation during the tetraanion formation stage. The solution becomes almost colorless.

4.4 UV-vis spectra of dissolved crystals

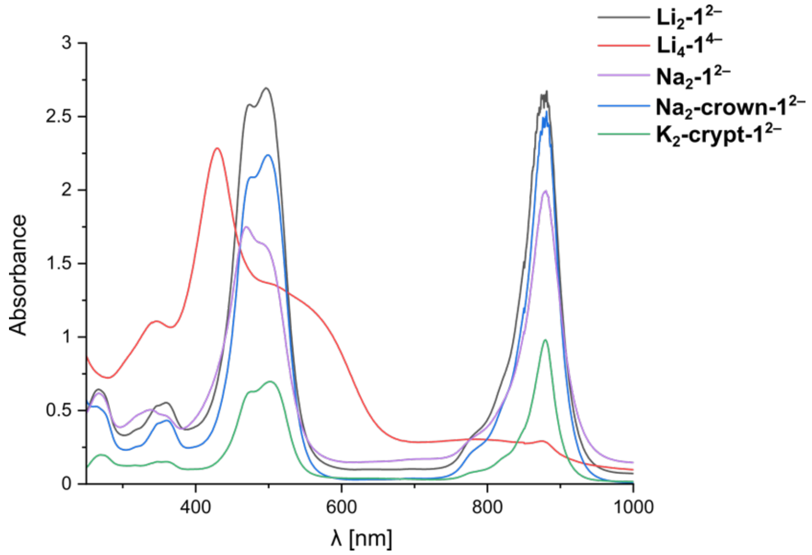

Figure S22. UV-vis-NIR spectra of crystals dissolved in THF. All spectra recorded in THF at 25 °C.

5. NMR spectroscopy

5.1 In-situ reaction of 1 with Li

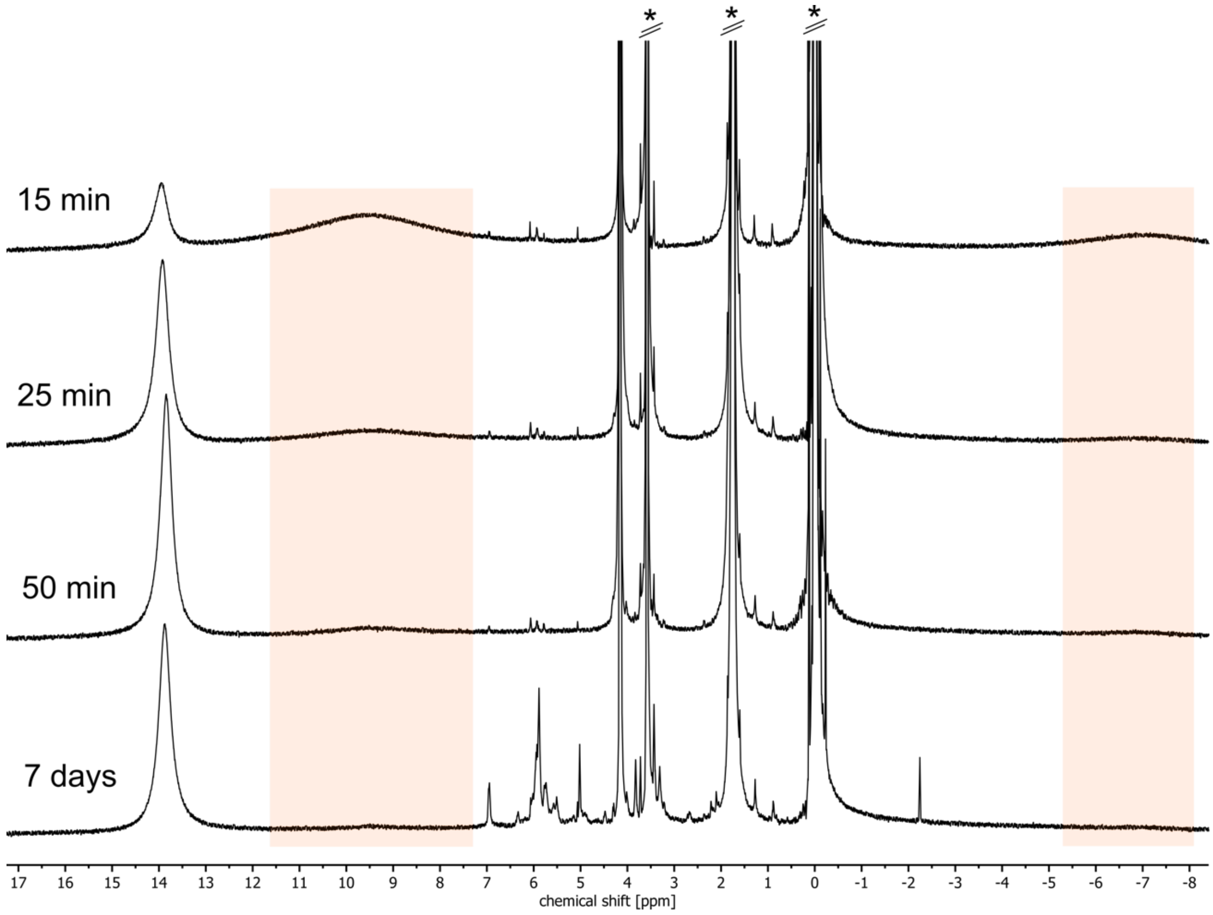

Figure S23. Reaction of **1** with Li monitored by ^{1}H NMR over time. Orange areas indicate region where dianion resonates. In this case, the reaction reached the stage of the dianion even before measurement was performed and tetraanion started to appear. After 50 min, there are almost no signs of the dianion in the mixture. Some decomposition/reactivity of impurity products started appearing after a week. THF-d_8, −30 °C, 500 MHz. Asterisk indicates signals of THF and TMS.

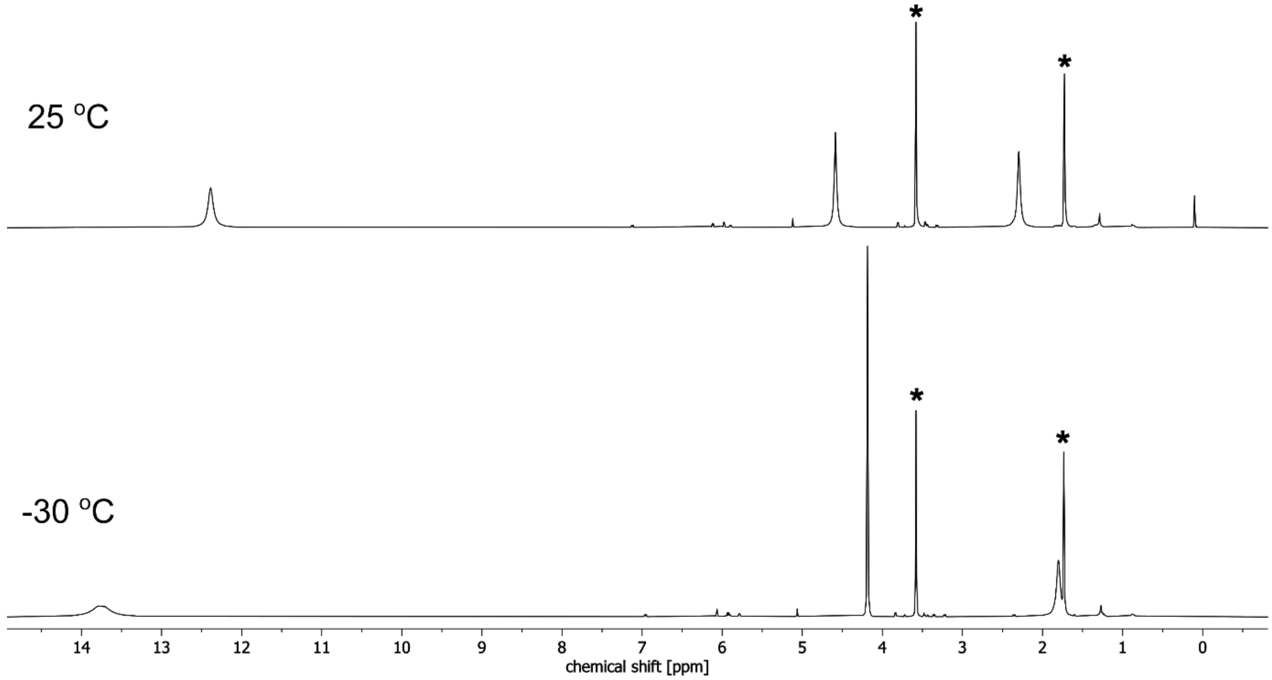

Figure S24. Temperature dependence of the ^{1}H NMR spectrum of the in-situ generated **Li$_4$-1^{4-}**. THF-d_8, 500 MHz. Asterisk indicates signals of THF. Intensity was normalized to the residual signal of THF. Note: different sample than in Fig. S23.

5.2 In-situ reaction of 1 with Na

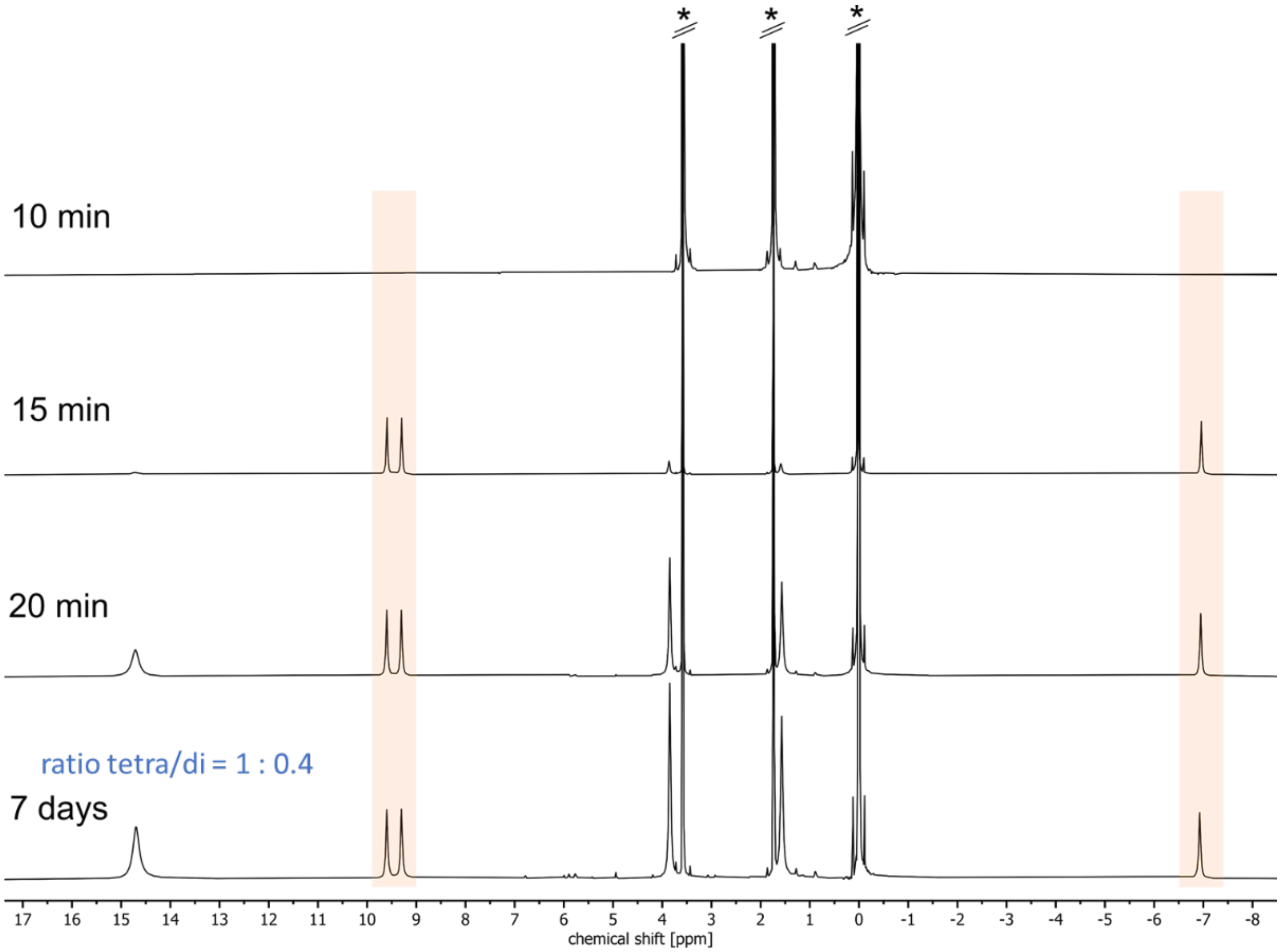

Figure S25. Reaction of **1** with Na monitored by ^{1}H NMR over time. Orange areas indicate region where dianion resonates. Mixture of dianion and tetraanion is formed. THF-d_8, −30 °C, 500 MHz. Asterisk indicates residual solvent signals of THF and signal of TMS.

5.3 Dissolved crystals of Li_2-$1^{2–}$

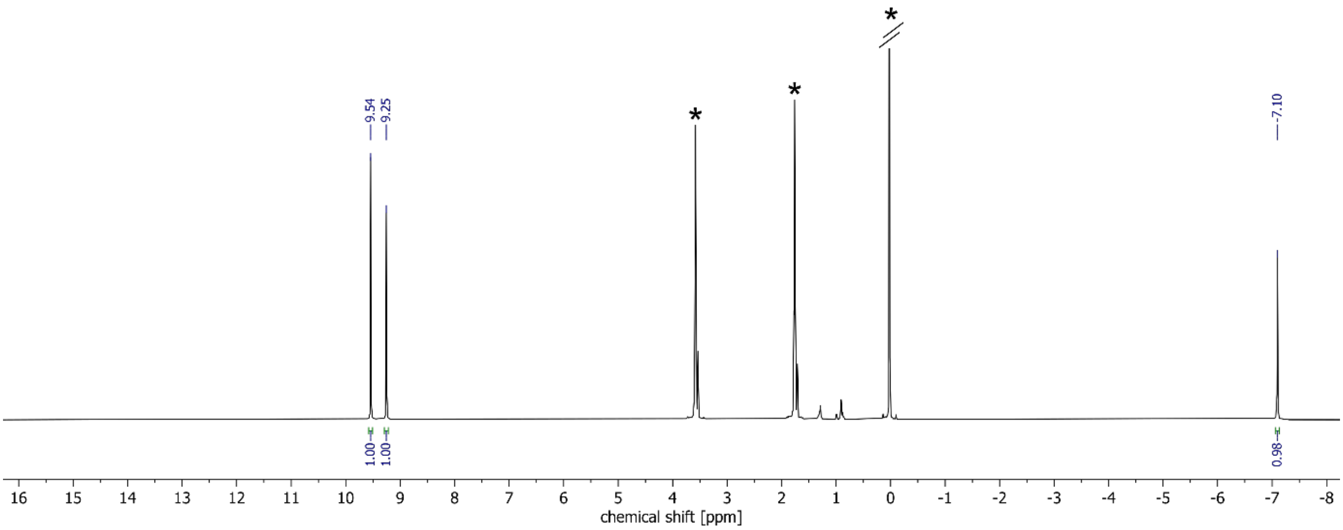

Figure S26. ^{1}H NMR spectrum of **Li₂-1²⁻**, THF-d_8, −30 °C, 500 MHz. Asterisk indicates residual solvent signals of THF and signal of TMS.

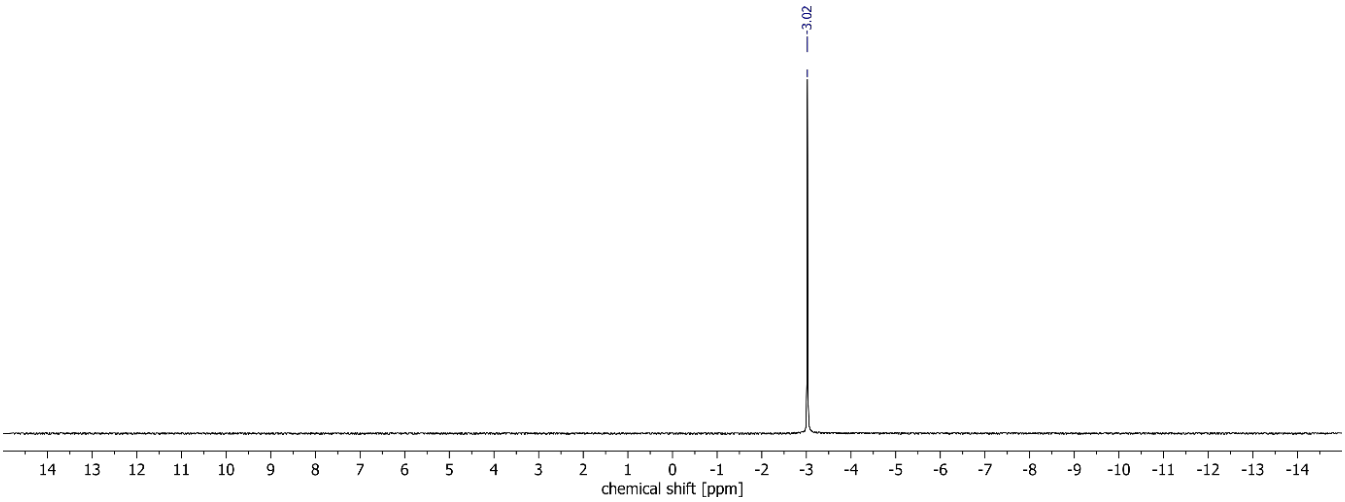

Figure S27. ^{7}Li NMR spectrum of **Li₂-1²⁻**, THF-d_8, −30 °C, 194 MHz.

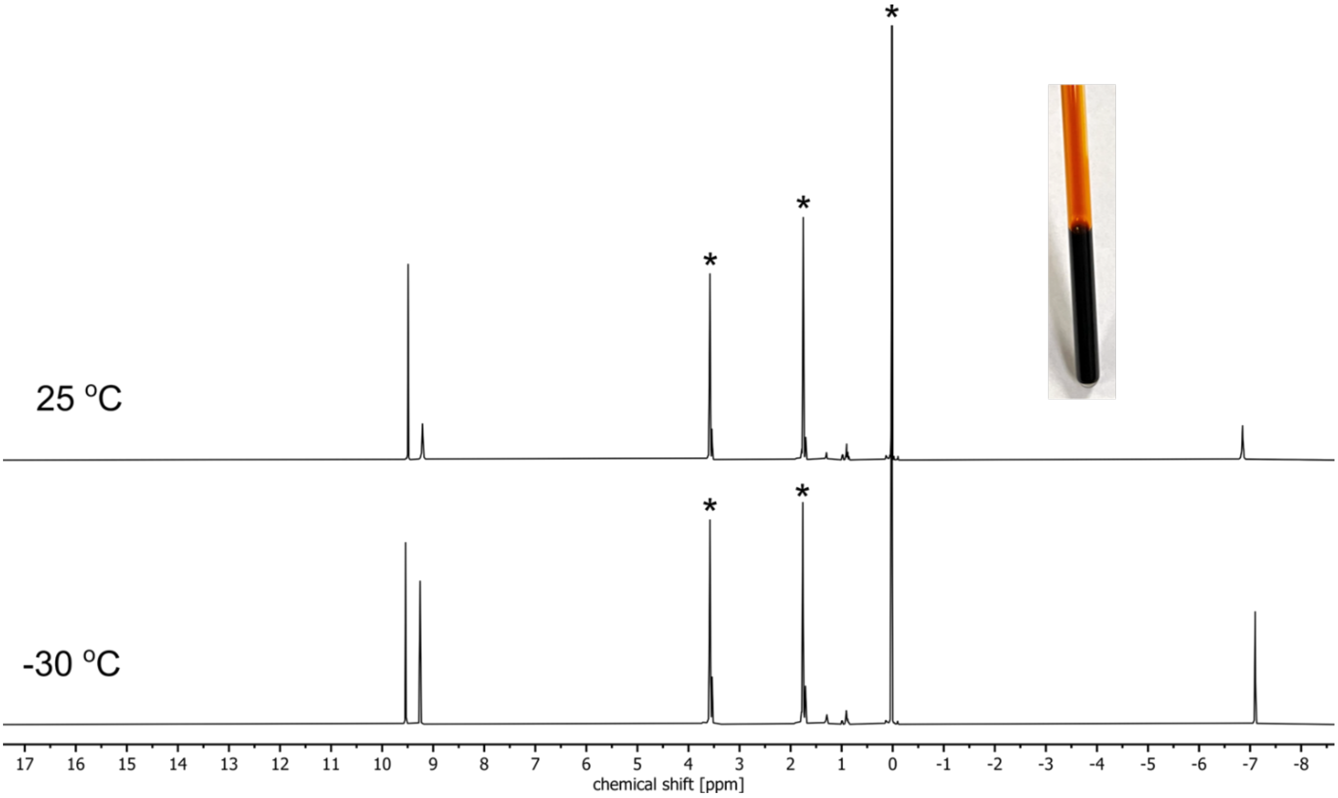

Figure S28. Comparison between ^{1}H NMR spectra of **Li_2-1^{2-}** at 25 °C (top) and −30 °C (bottom), THF-d_8, 500 MHz. Asterisk indicates residual solvent signals of THF and signal of TMS.

5.4 Dissolved crystals of Na_2-(6/7THF)-1^{2-}

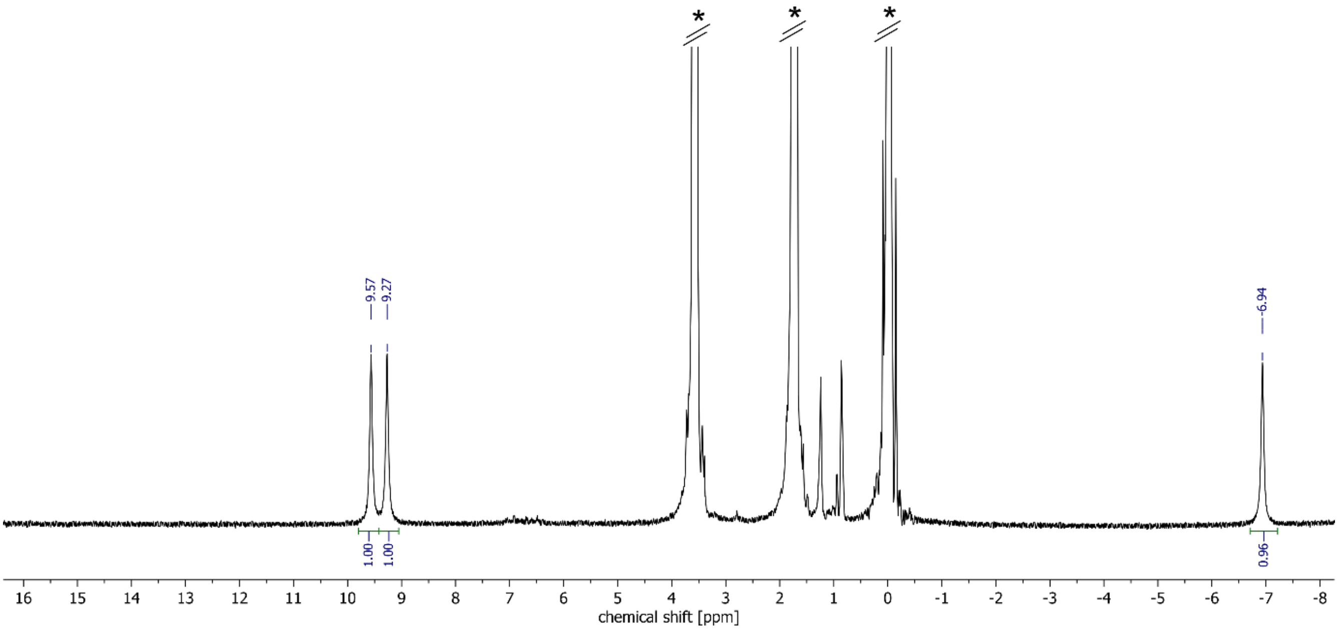

Figure S29. ^{1}H NMR spectrum of **Na_2-1^{2-}**, THF-d_8, −30 °C, 500 MHz. Asterisk indicates residual solvent signals of THF and signal of TMS.

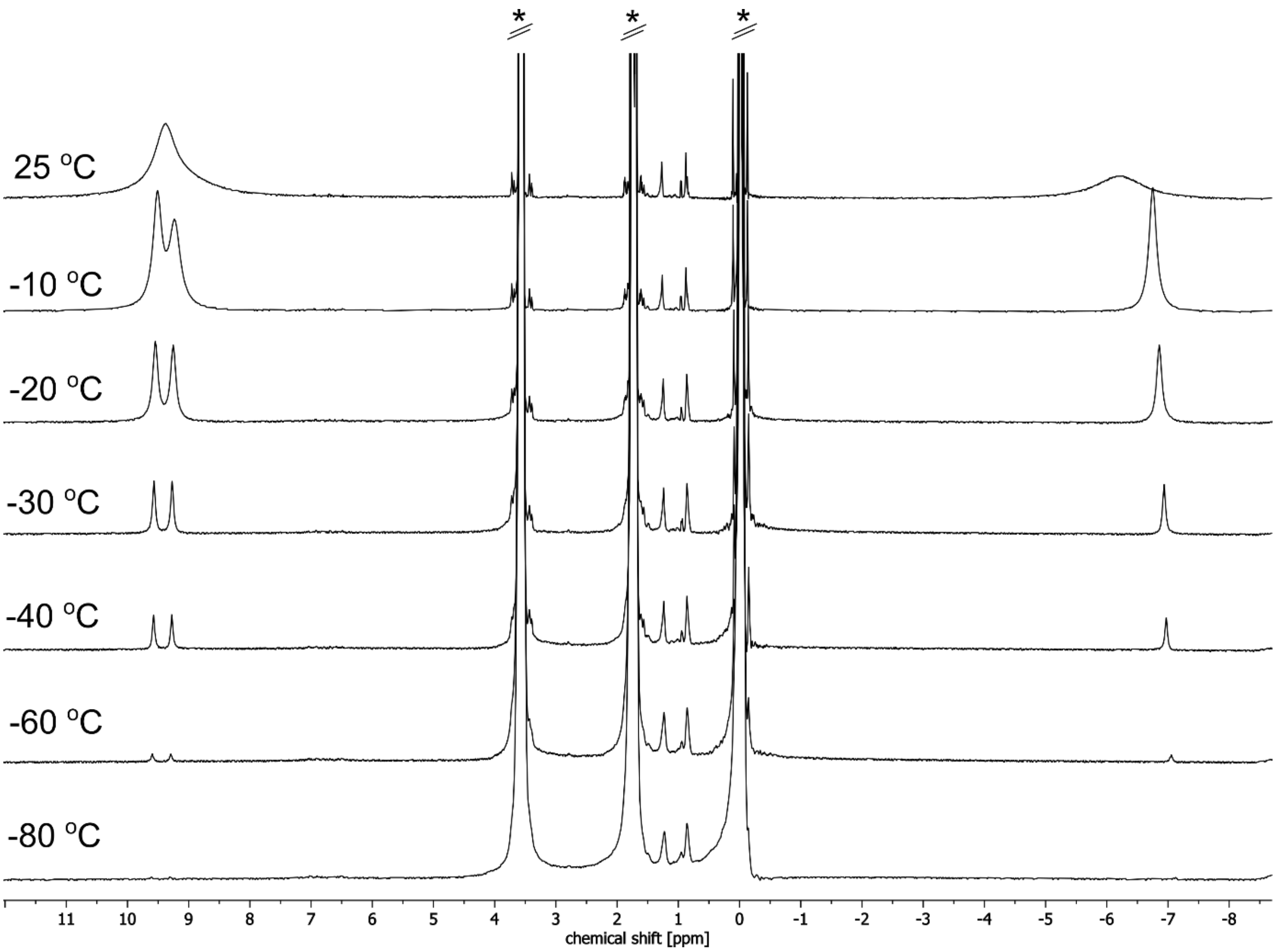

Figure S30. Comparison between ^{1}H NMR spectra of $\mathbf{Na_2\text{-}1^{2-}}$ at different temperatures, THF-d_8, 500 MHz. Asterisk indicates residual solvent signals of THF and signal of TMS.

5.5 Dissolved crystals of $\mathbf{Na_2\text{-}crown\text{-}1^{2-}}$

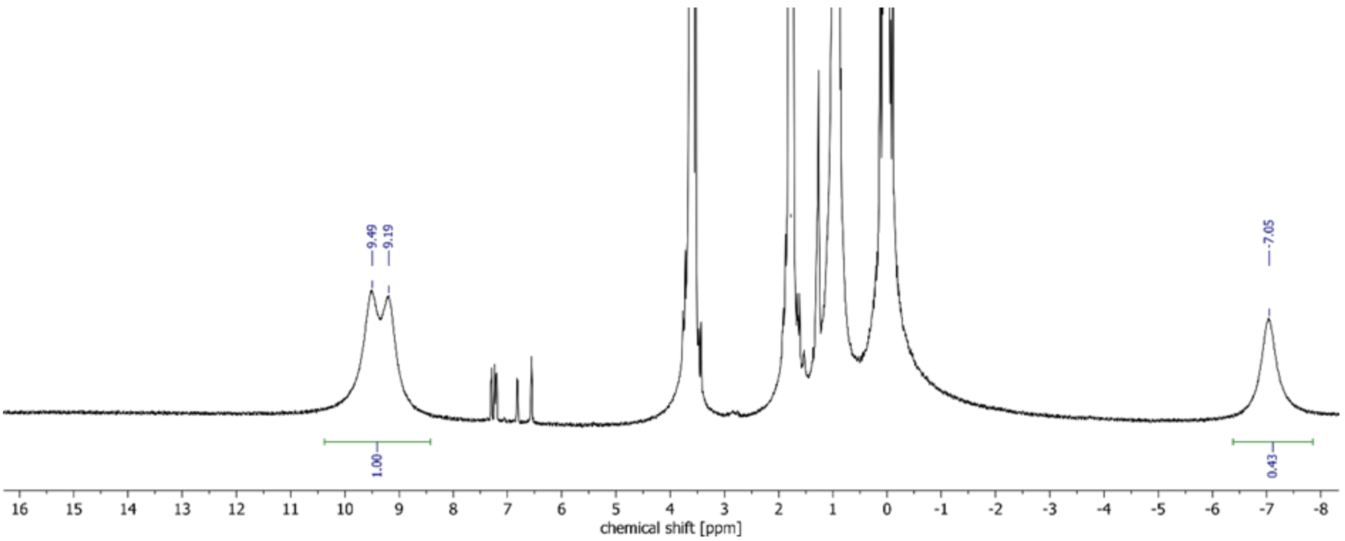

Figure S31. ^{1}H NMR spectrum of $\mathbf{Na_2\text{-}crown\text{-}1^{2-}}$, THF-$d_8$, −90 °C, 500 MHz. Solubility drops drastically at low temperatures and the sample crystallizes readily, that is why any impurity signals appear as similar in intensity to the product. Impurities come from reduced by-products after synthesis of **1** which could not be removed even by sublimation.

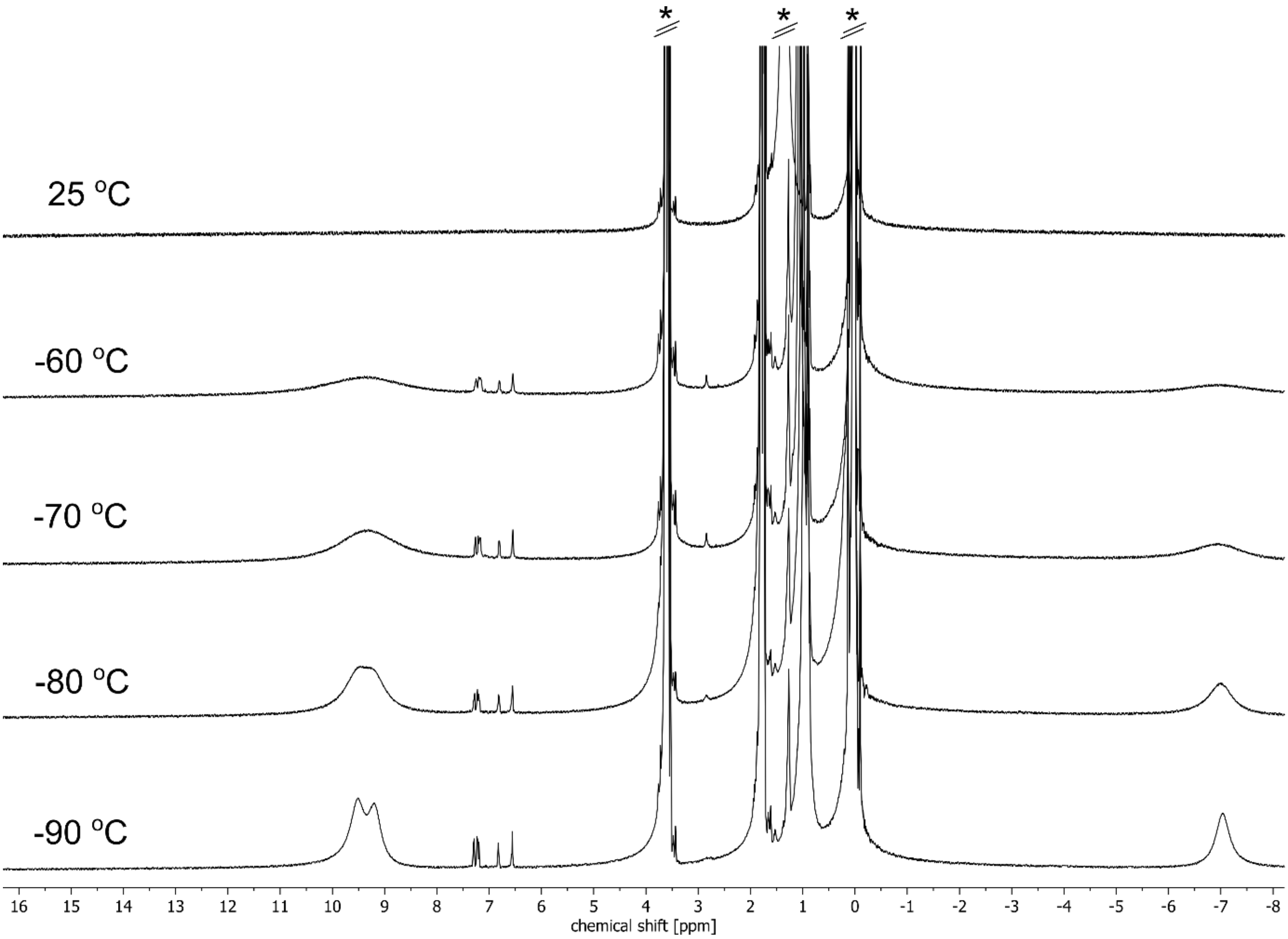

Figure S32. Temperature dependence of ^{1}H NMR spectrum of **Na$_2$-crown-1$^{2–}$**, THF-d_8, 500 MHz. Solubility drops drastically at low temperatures and the sample crystallizes readily, that is why any impurity signals appear as similar in intensity to the product. Impurities come from reduced by-products after synthesis of **1** which could not be removed even by sublimation. Asterisk indicates residual solvent signals of THF and signals of crown and TMS.

5.6 Dissolved crystals of K$_2$-cryptand-1$^{2–}$

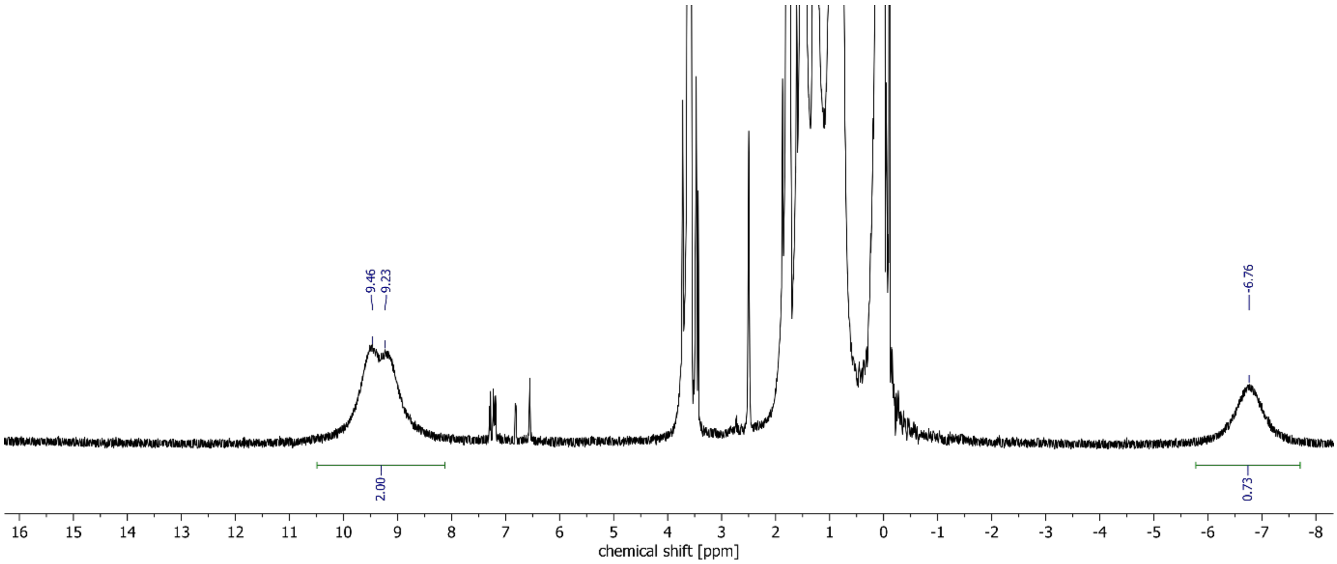

Figure S33. ^{1}H NMR spectrum of **K$_2$-crypt-1$^{2–}$**, THF-d_8, −80 °C, 500 MHz. Solubility drops drastically at low temperatures and the sample crystallizes readily, that is why any impurity signals appear as similar in intensity to the product. Impurities come from reduced by-products after synthesis of **1** which could not be removed even by sublimation.

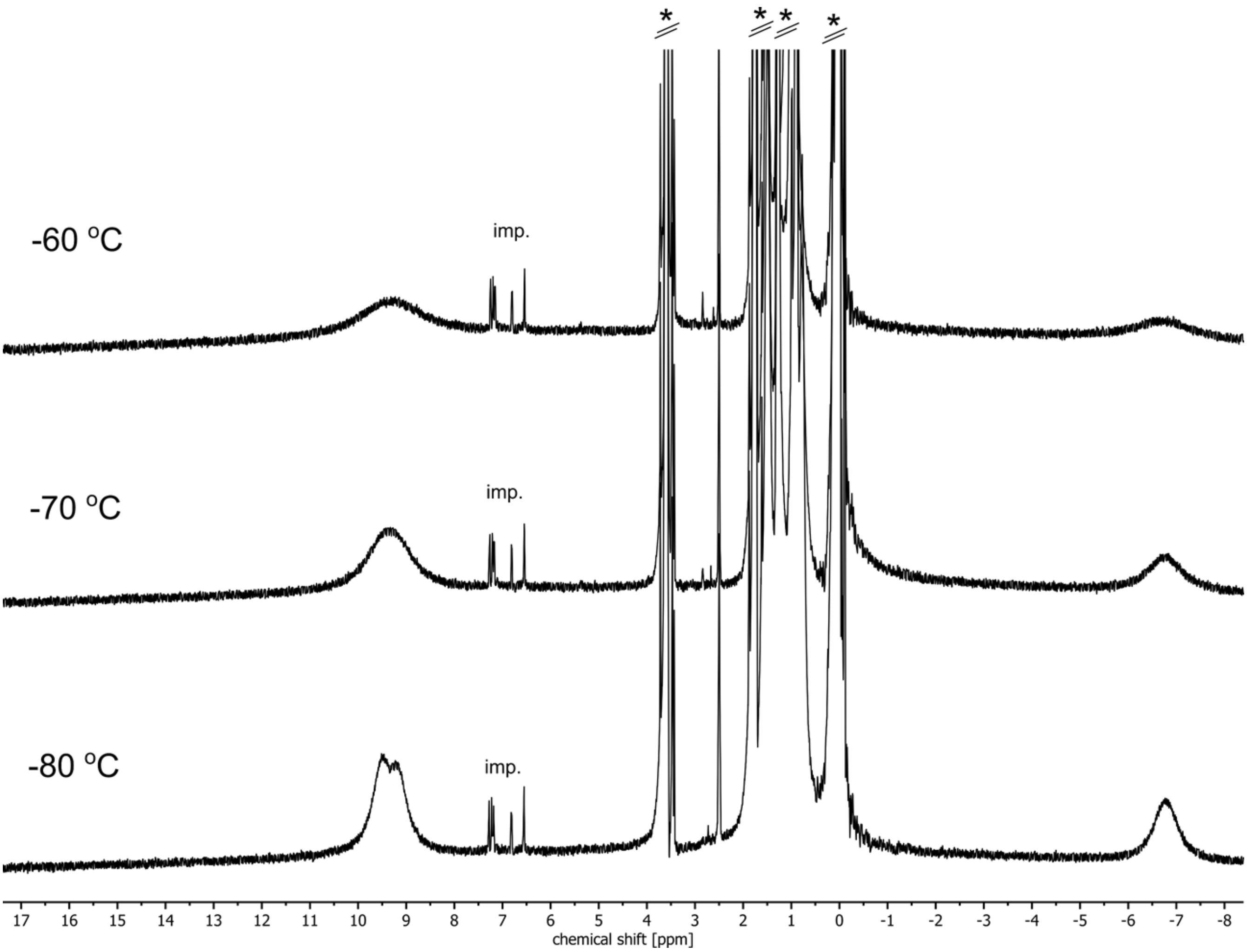

Figure S34. Temperature dependence of ^{1}H NMR spectrum of **K_2-crypt-1^{2-}**, THF-d_8, 500 MHz. Solubility drops drastically at low temperatures and the sample crystallizes readily, that is why any impurity signals appear as similar in intensity to the product. Impurities come from reduced by-products after synthesis of **1** which could not be removed even by sublimation. Asterisk indicates residual solvent signals of THF and signals of cryptand and TMS.

5.7 Dissolved crystals of Li_4-1^{4-}

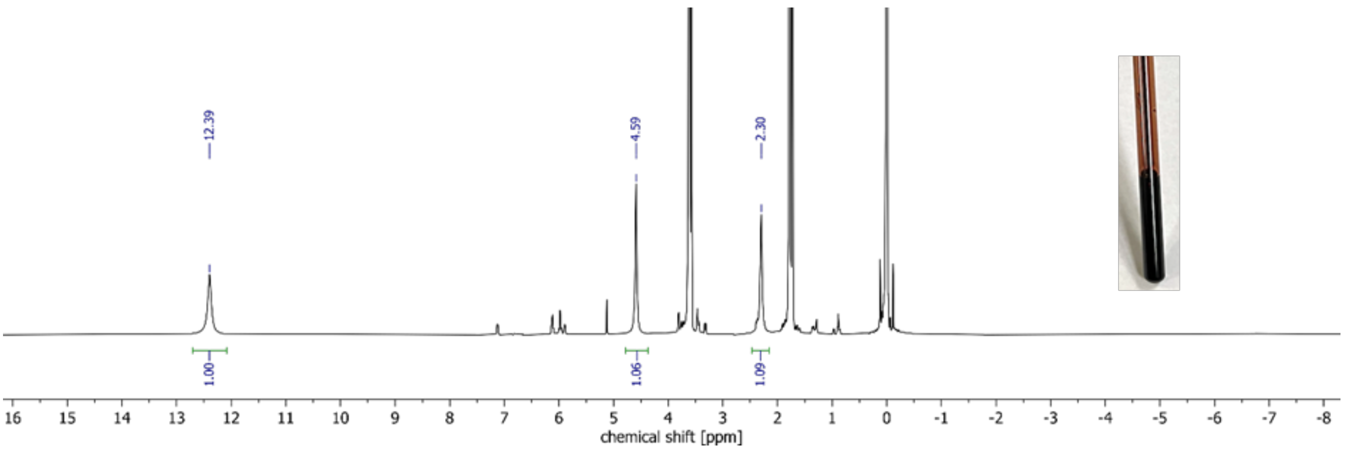

Figure S35. ^{1}H NMR spectrum of **Li_4-1^{4-}**, THF-d_8, 25 °C, 500 MHz.

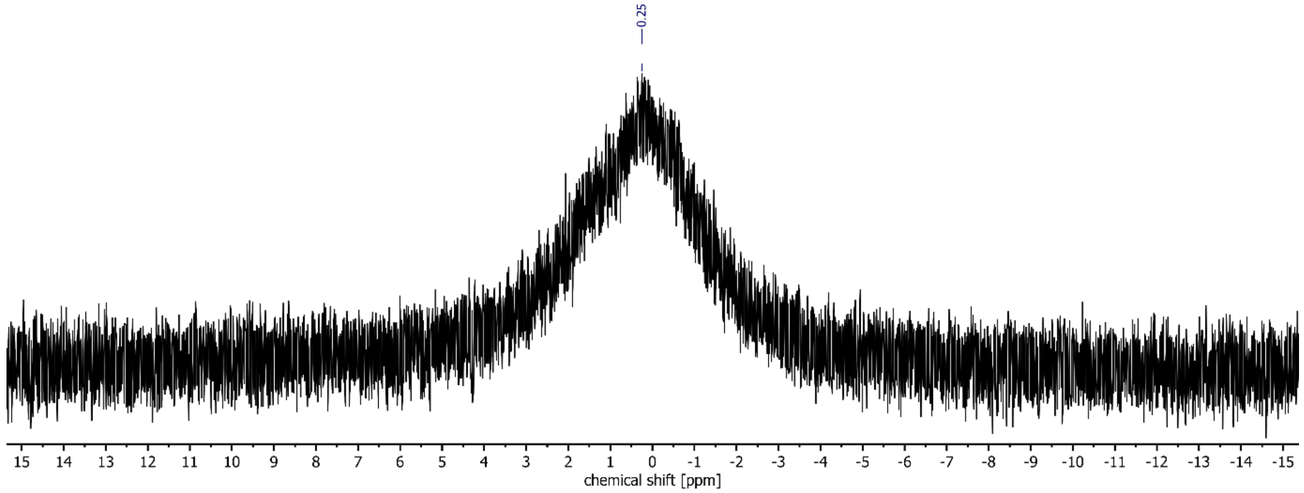

Figure S36. ^{7}Li NMR spectrum of **Li_4-1^{4-}**, THF-d_8, 25 °C, 194 MHz.

5.8 Reaction between 1 and Li in the presence of 15-crown-5

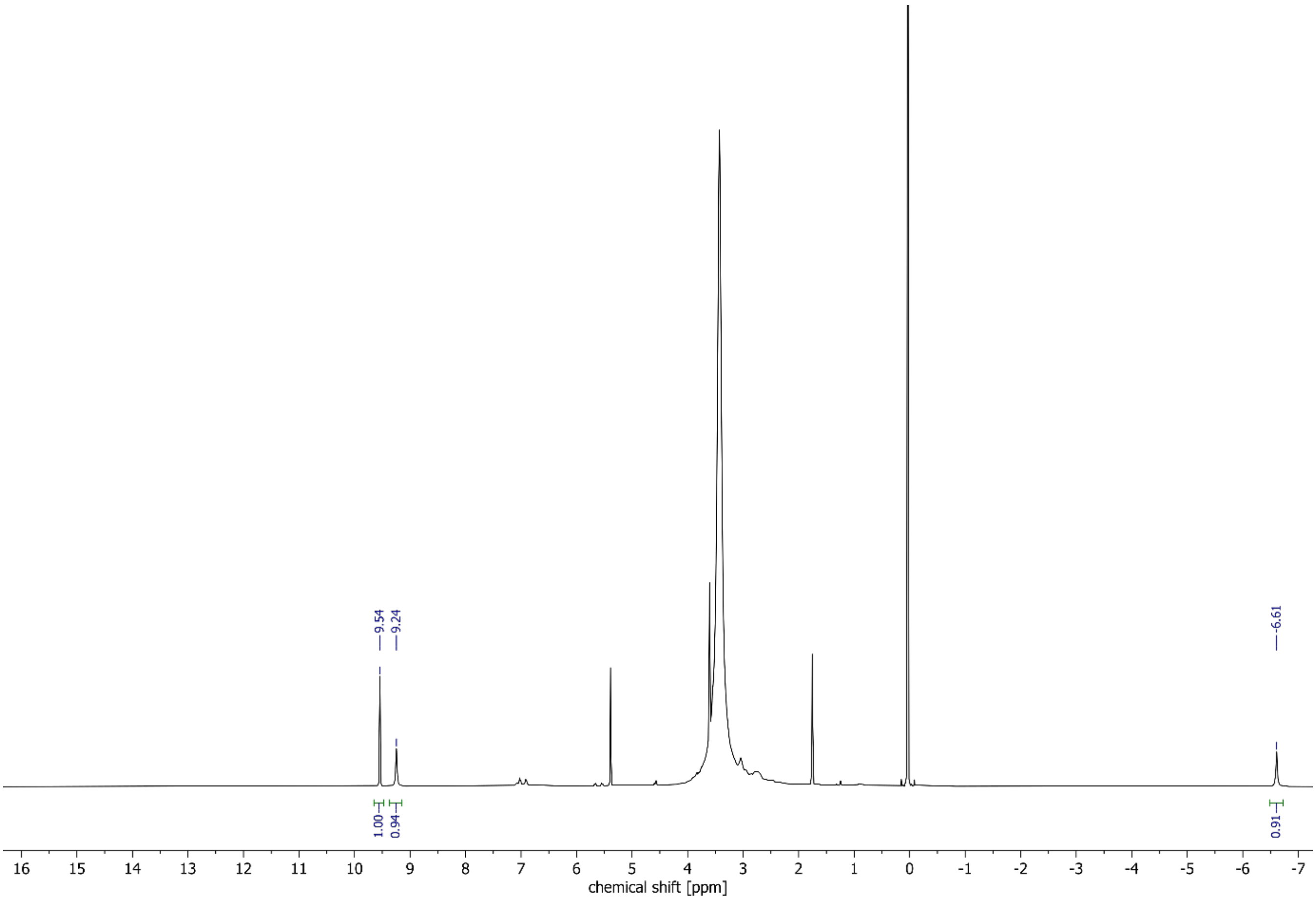

Figure S37. ^{1}H NMR spectrum of **Li_2-1^{2-}** containing excess of 15-crown-5, THF-d_8, 25 °C, 500 MHz.

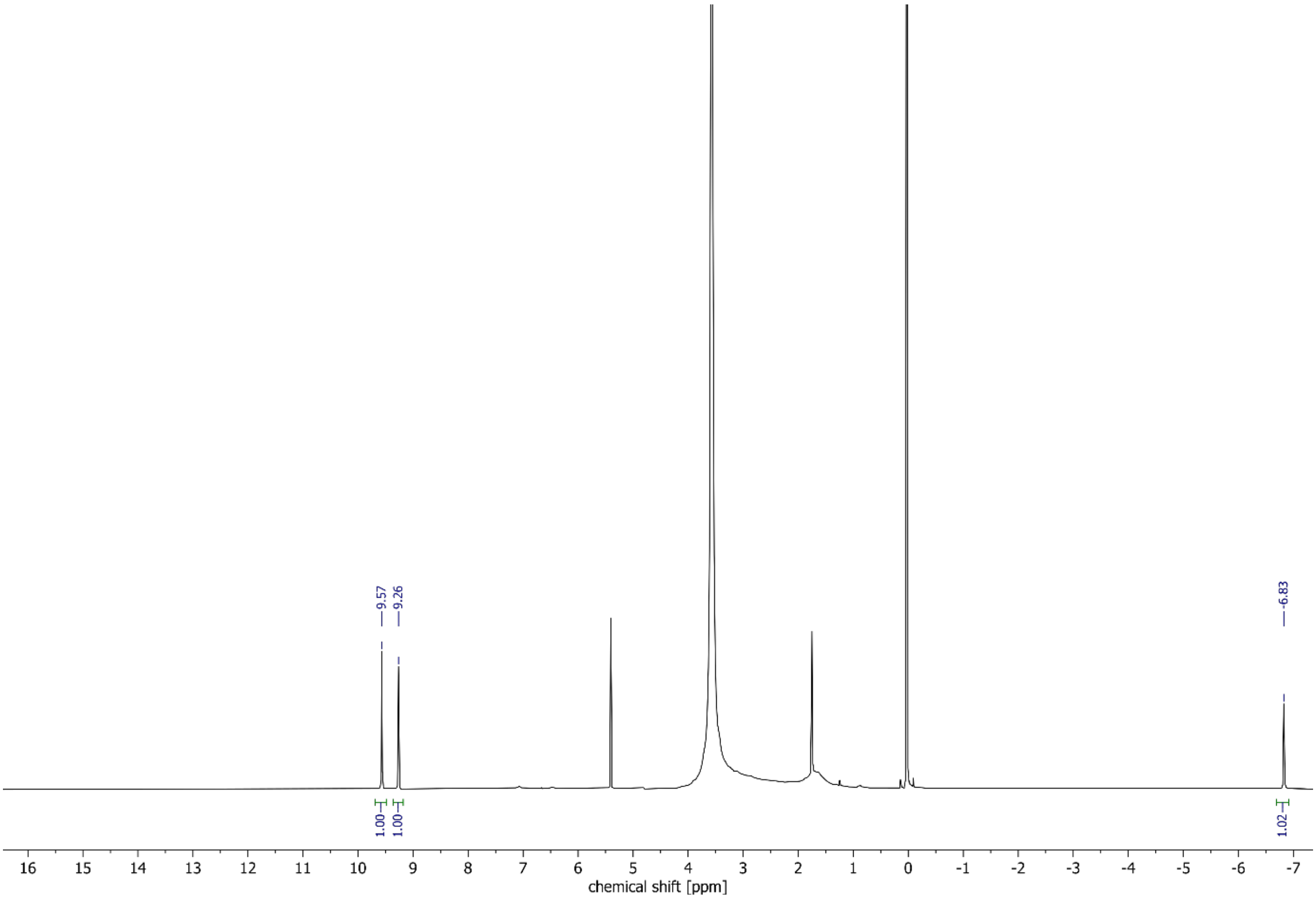

Figure S38. ^{1}H NMR spectrum of **Li_2-1^{2-}** containing excess of 15-crown-5, THF-d_8, −30 °C, 500 MHz.

6. Theoretical calculations

Geometries were optimized using Gaussian 16 software[9] on a CAM-B3LYP def2-TZVP level of theory in the gas phase (no solvation) with unconstrained C_1 symmetry.[10,11] We calculated all the vibrational frequencies and none of them were imaginary. TD-DFT calculations were performed similarly and using 64 states in the calculations, visualizing calculated UV-vis spectra using GaussView 6.0.16 software.

6.1. Calculated bond lengths and angles

Figure S39 shows the bond lengths in the linkers (black, outside the macro ring), in *para*-phenylenes (black, inside phenyls), torsion angles in linkers (blue and green) and dihedral angles between planes of *para*-phenylene rings (red). Dihedral and torsion angles were calculated using Mercury software. Torsion angles in the linkers have the same sign on opposite sides of the molecule (same torsion direction), the other two have the same sign but opposite. There are very small differences between calculated bond lengths around the molecule (second decimal place), the figure shows mean values for simplicity.

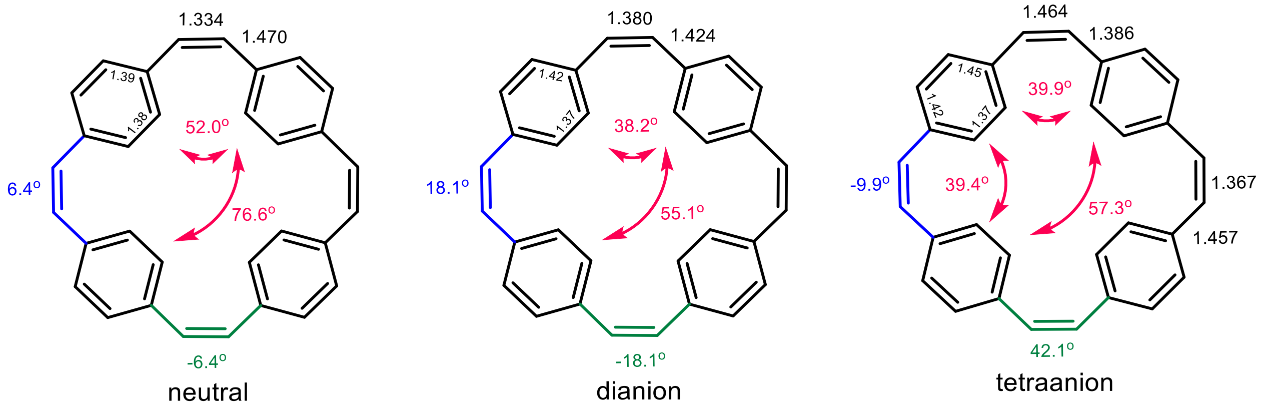

Figure S39. Structural parameters in geometry-optimized anions, CAM-B3LYP def2-TZVP.

6.2. Calculated Mulliken charges on atoms

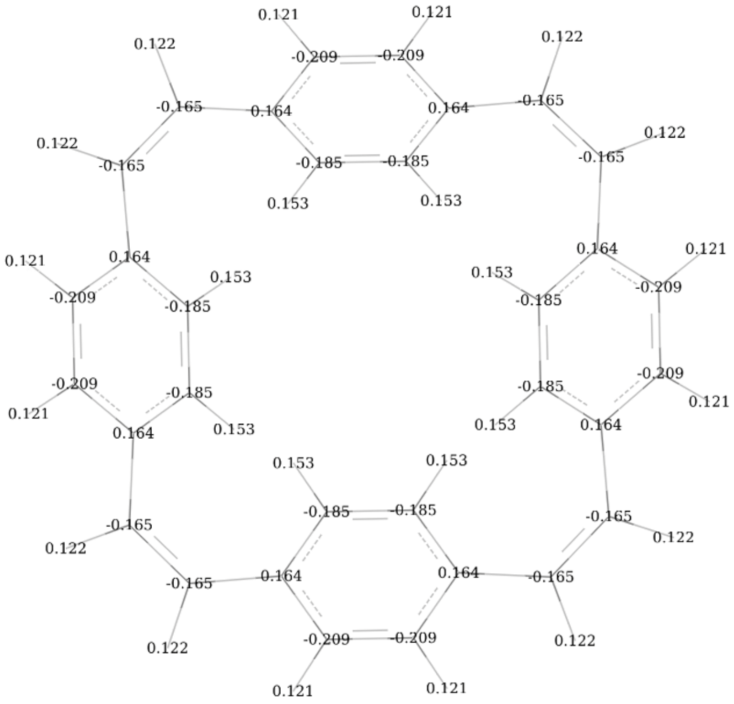

Figure S40. Mulliken charges calculated for atoms in neutral **1**.

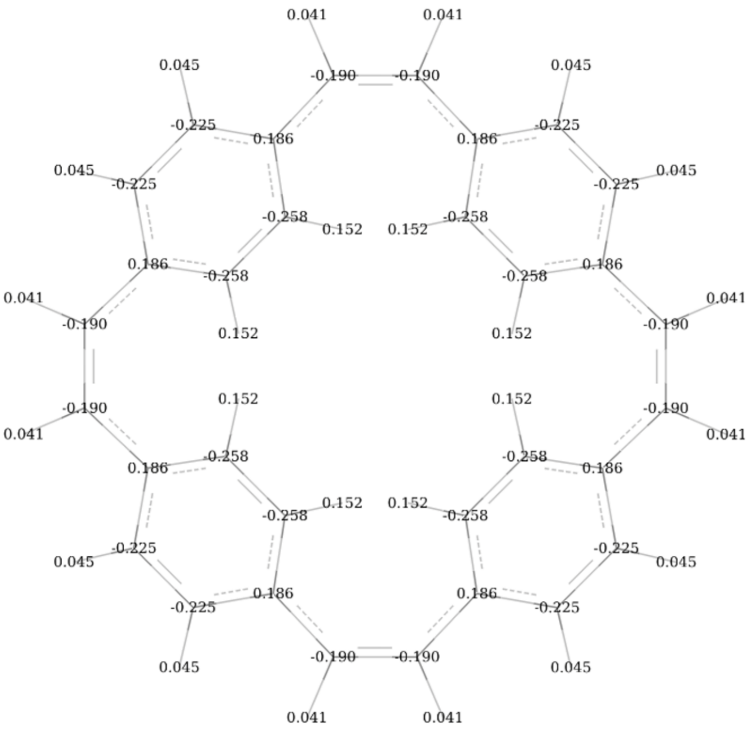

Figure S41. Mulliken charges calculated for atoms in $\mathbf{1}^{2-}$.

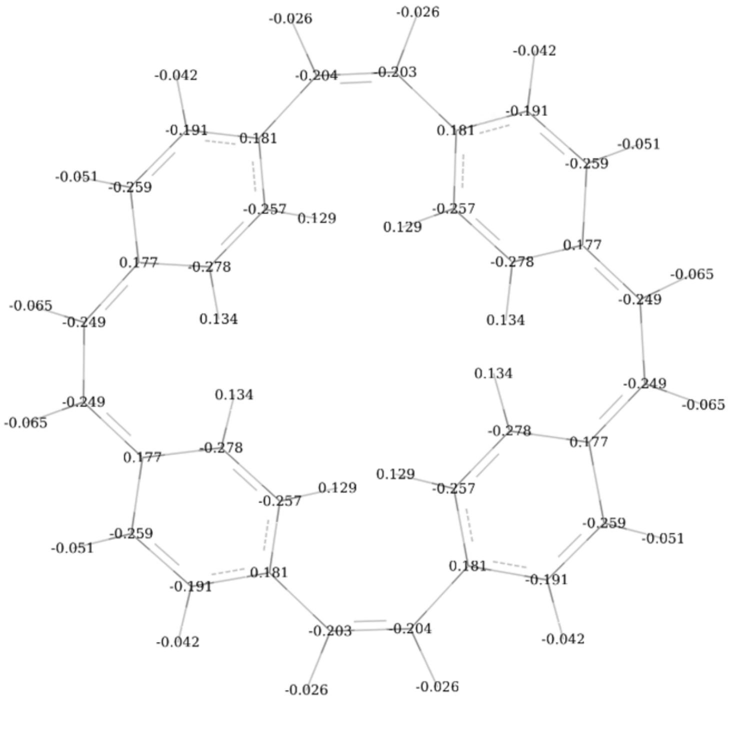

Figure S42. Mulliken charges calculated for atoms in $\mathbf{1}^{4-}$. Orientation corresponds to the one on Fig. S39.

6.3. Simulated UV-vis-NIR spectra

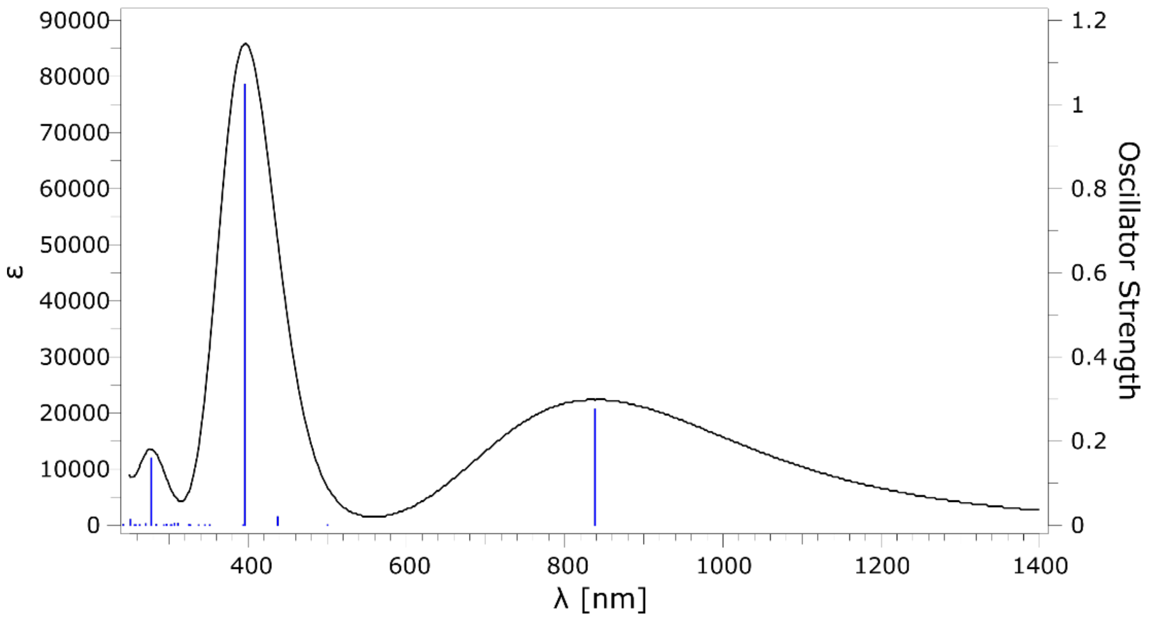

Figure S43. Simulated UV-vis-NIR spectrum of dianion. CAM-B3LYP def2-TZVP

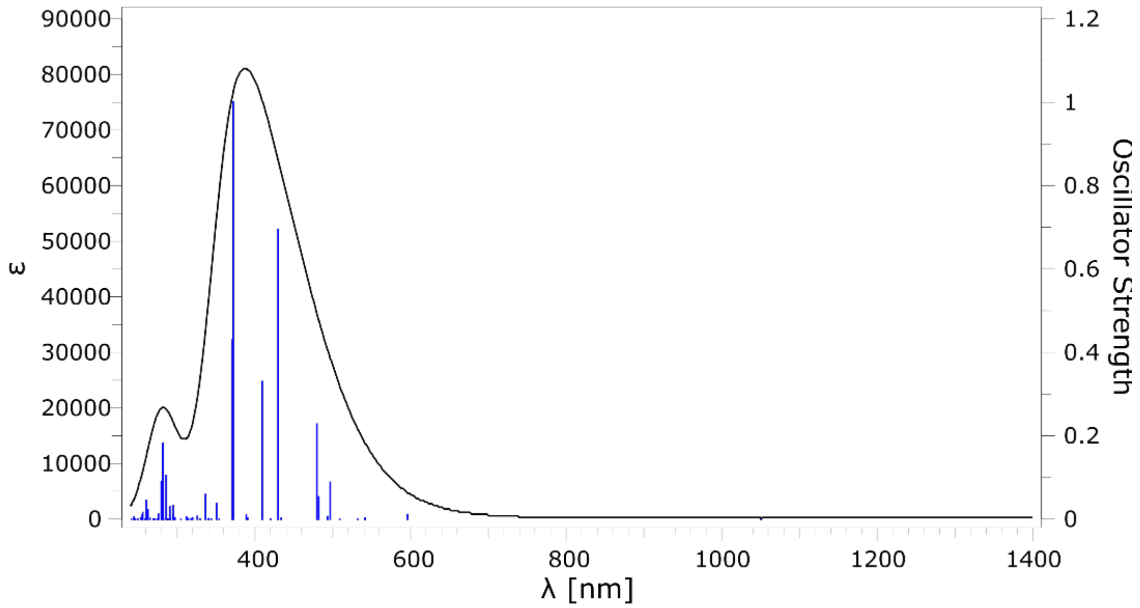

Figure S44. Simulated UV-vis spectrum of tetraanion. CAM-B3LYP def2-TZVP

6.4. Comparison of calculated and experimental geometries of di-anions.

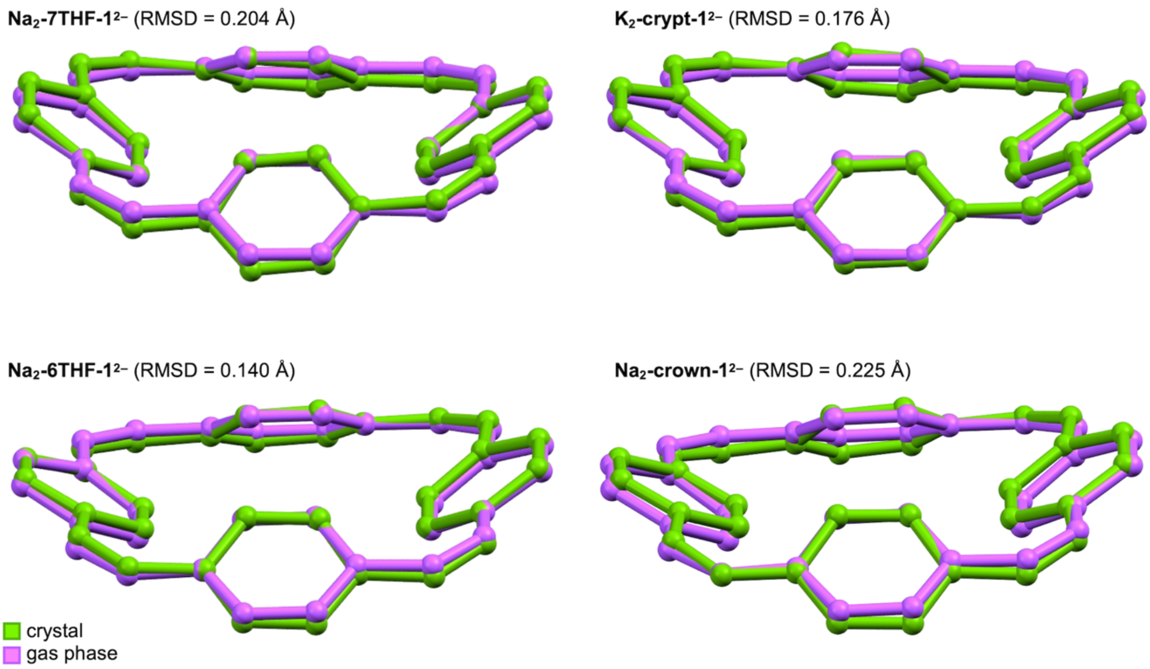

Figure S45. Overlay of crystallographic and calculated gas-phase structures **Na_2-7THF-$1^{2–}$**,**Na_2-6THF-$1^{2–}$**, **K_2-crypt-$1^{2–}$** and **Na_2-crown-$1^{2–}$** (with RMSDs between experimental and calculated structures). Note that the counter cations were not included in the gas phase DFT calculations. Calculations: CAM-B3LYP def2-TZVP.

6.5. Cartesian coordinates

Cartesian coordinates of the calculated gas-phase molecular geometries of **1**, **$1^{2–}$** and **$1^{4–}$** are available from the Zenodo public repository (https://zenodo.org/record/8239309 and https://doi.org/10.5281/zenodo.8239309).

6.6. NICS calculations

Positions of dummy atoms (Bq) were calculated at the centroids of the macrocycle, phenyl rings and at the middle of the C=C bonds in the linkers. They were also calculated 1 Å above and below these points (Fig. S45). The *Z* axis (direction of the magnetic field) is perpendicular to the macrocycle plane. NICS analysis was reported for the neutral **1** and dianion **$1^{2–}$** but has never been reported before for the tetraanion **$1^{4–}$**.[12] In some cases, the NICS(1) and NICS(–1) values on the opposite sides of the ring are the same (equivalent due to symmetry) and were indicated as such in the Table S2. In phenyl rings, NICS(1) / NICS(–1) were indicated as *in*/*out* to distinguish between Bq positions pointing towards the inside the macroring (*in*) or towards outside of the macroring (*out*).

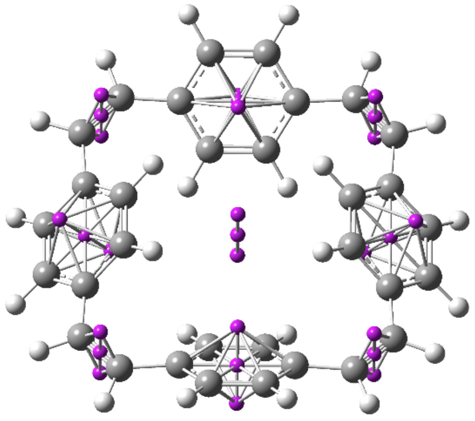

Figure S46. Location of dummy atoms (violet) used for NICS calculations (for **1** as an example).

Table S16. Calculated NICS values.

		NICS(0)		NICS (1, in)		NICS(-1, out)	
		iso	zz	iso	zz	iso	zz
**1**	middle	-0.39	8.82	-0.20	7.14	equiv.	equiv.
	linker	-42.43	-51.33	-11.55	-15.16	equiv.	equiv.
	phenyl	-6.68	-4.87	-7.64	-10.50	-9.11	-14.18
**1**$^{2-}$	middle	-15.51	-39.99	-14.10	-37.71	equiv.	equiv.
	linker	-53.58	-78.51	-19.91	-41.91	equiv.	equiv.
	phenyl	-9.16	-16.01	-14.91	-40.33	-6.20	-13.60
**1**$^{4-}$	middle	9.31	33.22	8.28	28.48	equiv.	equiv.
	phenyl	5.33	24.46	5.40	17.14	0.23	2.92
	linker, longer	-41.63	-35.16	-5.40	-0.78	equiv.	equiv.
	linker, shorter	-41.00	-32.52	-5.65	2.36	equiv.	equiv.

7. HOMA Calculations

HOMA (Harmonic Oscillator Model of Aromaticity) is one of the simplest and most widely used indices for describing aromaticity based on molecular geometry. It uses the C-C bond length in benzene as a standard of perfect aromaticity. The HOMA index can be calculated using the following equation:[13]

$$\mathrm{HOMA} = 1 - \frac{\alpha}{n}\sum_{i=1}^{n}(R_i - R_{\mathrm{opt}})^2$$

where R_i and R_{opt} are the ith bond length of the C-C bond in the analyzed ring and the bond length of benzene ring (R_{opt} = 1.388 Å), respectively. n is the number of C-C bonds in the analyzed ring and α = 257.7 $Å^{-2}$ is a normalization factor that gives HOMA value of 1 for perfect aromatic benzene ring and a HOMA value of 0 for an alternating nonaromatic Kekulé cyclohexatriene ring.

The bond length data and calculated HOMA indexes of the vinylene bridges in **1**, $\mathbf{1}^{2-}$ and $\mathbf{1}^{4-}$ are listed in the Table S17:

Table S17. Bond length data used for calculating HOMA values of vinylene bridges in **1**, $\mathbf{1}^{2-}$ and $\mathbf{1}^{4-}$.

**Molecule**	**Bond lengths of vinylene bridges (C-CH=CH-C) (Å)**	**HOMA**
**1** neutral (DFT)	1.4703, 1.3342, 1.4703	–0.41
**1** neutral (crystal structure)[8]	1.475(5), 1.306(5), 1.471(5), 1.464(5), 1.313(5), 1.454(5), 1.467(5), 1.317(5), 1.485(5), 1.462(5), 1.310(5), 1.478(5) [8]	–0.66
$\mathbf{1}^{2-}$ (DFT)	1.4242, 1.3803, 1.4243	0.77
$\mathbf{Li_2\text{-}1^{2-}}$ (crystal structure)	1.427(2), 1.386(2), 1.430(2), 1.421(2), 1.402(2), 1.428(2), 1.423(2), 1.383(2), 1.435(2), 1.441(2), 1.374(2), 1.438(2)	0.67
$\mathbf{Na_2\text{-}6THF\text{-}1^{2-}}$ (crystal structure)	1.4354(13), 1.3906(16), 1.4307(15), 1.4311(15), 1.3831(16), 1.4302(13), 1.4302(13), 1.3947(14), 1.4272(13), 1.4283(13), 1.3823(14), 1.4311(13)	0.69
$\mathbf{Na_2\text{-}7THF\text{-}1^{2-}}$ (crystal structure)	1.4313(5), 1.3967(5), 1.4347(4), 1.4306(4), 1.3876(5), 1.4319(5), 1.4324(5), 1.3946(5), 1.4339(4), 1.4316(4), 1.3862(5), 1.4327(5)	0.66
$\mathbf{Na_2\text{-}crown\text{-}1^{2-}}$ (crystal structure)	1.435(7), 1.396(8), 1.413(8), 1.403(11), 1.399(12), 1.423(8), 1.429(7), 1.388(6), 1.422(6), 1.425(7), 1.373(7), 1.448(7)	0.73
$\mathbf{K_2\text{-}crypt\text{-}1^{2-}}$ (crystal structure)	1.441(10), 1.352(7), 1.414(9), 1.418(10), 1.387(13), 1.441(14), 1.434(17), 1.378(10), 1.414(15), 1.451(17), 1.371(9), 1.406(10)	0.66
$\mathbf{1}^{4-}$ (DFT)	1.3851, 1.4640, 1.3851, 1.4560, 1.3665, 1.4567	0.33
$\mathbf{Li_4\text{-}1^{4-}}$ (crystal structure)	1.405(4), 1.469(4), 1.413(4), 1.466(4), 1.351(5), 1.458(4), 1.411(4), 1.476(4), 1.422(4), 1.464(4), 1.351(4), 1.457(4)	0.12

8. References

1 N. V. Kozhemyakina, J. Nuss and M. Jansen, *Z. Anorg. Allg. Chem.,* 2009, **635***,* 1355–1361.

2 S. Eder, D.-J. Yoo, W. Nogala, M. Pletzer, A. Santana Bonilla, A. J. P. White, K. E. Jelfs, M. Heeney, J. W. Choi and F. Glöcklhofer, *Angew. Chem. Int. Ed.,* 2020, **59**, 12958–12964.

3 SAINT; part of Bruker APEX3 software package (version 2017.3-0): Bruker AXS, 2017.

4 SADABS; part of Bruker APEX3 software package (version 2017.3-0): Bruker AXS, 2017.

5 G. M. Sheldrick, *Acta Crystallogr.,* 2015, **A71**, 3–8.

6 G. M. Sheldrick, *Acta Crystallogr.,* 2015, **C71**, 3–8.

7 O. V. Dolomanov, L. J. Bourhis, R. J. Gildea, J. A. K. Howard and H. Puschmann, *J. Appl. Crystallogr.,* 2009, **42**, 339–341.

8 E. Ljungström, O. Lindqvist and O. Wennerström, *Acta Cryst B,* 1978, **34**, 1889–1893.

9 Gaussian 16, Revision C.01, M. J. Frisch, G. W. Trucks, H. B. Schlegel, G. E. Scuseria, M. A. Robb, J. R. Cheeseman, G. Scalmani, V. Barone, G. A. Petersson, H. Nakatsuji, X. Li, M. Caricato, A. V. Marenich, J. Bloino, B. G. Janesko, R. Gomperts, B. Mennucci, H. P. Hratchian, J. V. Ortiz, A. F. Izmaylov, J. L. Sonnenberg, D. Williams-Young, F. Ding, F. Lipparini, F. Egidi, J. Goings, B. Peng, A. Petrone, T. Henderson, D. Ranasinghe, V. G. Zakrzewski, J. Gao, N. Rega, G. Zheng, W. Liang, M. Hada, M. Ehara, K. Toyota, R. Fukuda, J. Hasegawa, M. Ishida, T. Nakajima, Y. Honda, O. Kitao, H. Nakai, T. Vreven, K. Throssell, J. A. Montgomery, Jr., J. E. Peralta, F. Ogliaro, M. J. Bearpark, J. J. Heyd, E. N. Brothers, K. N. Kudin, V. N. Staroverov, T. A. Keith, R. Kobayashi, J. Normand, K. Raghavachari, A. P. Rendell, J. C. Burant, S. S. Iyengar, J. Tomasi, M. Cossi, J. M. Millam, M. Klene, C. Adamo, R. Cammi, J. W. Ochterski, R. L. Martin, K. Morokuma, O. Farkas, J. B. Foresman and D. J. Fox, Gaussian, Inc., Wallingford CT, 2016.

10 T. Yanai, D. P. Tew and N. C. Handy, *Chem. Phys. Lett.,* 2004, **393**, 51–57.

11 F. Weigend and F. Ahlrichs, *Phys. Chem. Chem. Phys.,* 2005, **7**, 3297–3305.

12 F. Plasser and F. Glöcklhofer, *Eur. J. Org. Chem.,* 2021, **2021**, 2529–2539.

13 a) J. Kruszewski and T. M. Krygowski, *Tetrahedron Lett.,* 1972, **13**, 3839–3842; b) T. M. Krygowski and M. K. Cyrański, *Chem. Rev.,* 2001, **101**, 1385–1419; c) M. K. Cyrański, *Chem. Rev.,* **2005**, *105*, 3773–3811; d) T. M. Krygowski, H. Szatylowicz, O. A. Stasyuk, J. Dominikowska and M. Palusiak, *Chem. Rev.,* 2014, **114**, 6383–6422; e) M. Makino, N. Nishina and J. Aihara, *J. Phys. Org. Chem.,* 2018, **31**, e3783.
